# Modulation of the β-Catenin Signaling Pathway by the Dishevelled-Associated Protein Hipk1

**DOI:** 10.1371/journal.pone.0004310

**Published:** 2009-02-02

**Authors:** Sarah H. Louie, Xiao Yong Yang, William H. Conrad, Jeanot Muster, Stephane Angers, Randall T. Moon, Benjamin N. R. Cheyette

**Affiliations:** 1 Howard Hughes Medical Institute, Department of Pharmacology, and Institute for Stem Cell and Regenerative Medicine, University of Washington School of Medicine, Seattle, Washington, United States of America; 2 Department of Psychiatry, and Graduate Program in Developmental Biology, Program in Biological Sciences, University of California San Francisco, San Francisco, California, United States of America; Max Planck Institute of Molecular Cell Biology and Genetics, Germany

## Abstract

**Background:**

Wnts are evolutionarily conserved ligands that signal through β-catenin-dependent and β-catenin–independent pathways to regulate cell fate, proliferation, polarity, and movements during vertebrate development. *Dishevelled* (*Dsh/Dvl*) is a multi-domain scaffold protein required for virtually all known Wnt signaling activities, raising interest in the identification and functions of Dsh-associated proteins.

**Methodology:**

We conducted a yeast-2-hybrid screen using an N-terminal fragment of Dsh, resulting in isolation of the *Xenopus laevis* ortholog of Hipk1. Interaction between the Dsh and Hipk1 proteins was confirmed by co-immunoprecipitation assays and mass spectrometry, and further experiments suggest that Hipk1 also complexes with the transcription factor Tcf3. Supporting a nuclear function during *X. laevis* development, Myc-tagged Hipk1 localizes primarily to the nucleus in animal cap explants, and the endogenous transcript is strongly expressed during gastrula and neurula stages. Experimental manipulations of Hipk1 levels indicate that Hipk1 can repress Wnt/β-catenin target gene activation, as demonstrated by β-catenin reporter assays in human embryonic kidney cells and by indicators of dorsal specification in *X. laevis* embryos at the late blastula stage. In addition, a subset of Wnt-responsive genes subsequently requires Hipk1 for activation in the involuting mesoderm during gastrulation. Moreover, either over-expression or knock-down of Hipk1 leads to perturbed convergent extension cell movements involved in both gastrulation and neural tube closure.

**Conclusions:**

These results suggest that Hipk1 contributes in a complex fashion to Dsh-dependent signaling activities during early vertebrate development. This includes regulating the transcription of Wnt/β-catenin target genes in the nucleus, possibly in both repressive and activating ways under changing developmental contexts. This regulation is required to modulate gene expression and cell movements that are essential for gastrulation.

## Introduction

Gastrulation is a critical process during early vertebrate development involving changes in cell fate and cell behavior to generate the three germ layers of the embryo [1]. Wnts are evolutionarily conserved extracellular glycoproteins required for regulation of these cell fates and behaviors [2], [3]. Wnts function by activating receptor-mediated signal transduction pathways, notably the Wnt/β-catenin pathway and the β-catenin-independent Wnt/Ca^++^ and Planar Cell Polarity (PCP) pathways [4]–[7]. All these pathways involve Wnt ligands binding to Frizzled receptor complexes, and it is likely that the presence or absence of Lrp5/6 co-receptors [8] differentially stimulates the intracellular phosphoprotein Dishevelled (Dsh) to signal downstream through distinct biochemical pathways [9]–[17].

Linkage of Dsh to the β-catenin pathway involves regulated changes in Dsh stability, phosphorylation, and protein interactions [18]–[20]. The net result of these changes is to reduce degradation of non-membrane-associated β-catenin, leading to its accumulation in the nucleus and modulation of gene transcription through interactions with the Lef/Tcf family of transcription factors [21]–[23]. In contrast, the PCP and potentially related Wnt/Ca^++^ pathways involve Dsh signaling through β-catenin-independent mechanisms. The PCP and Wnt/Ca^++^ pathways have many downstream effectors, such as Ca^++^-sensitive enzymes, small GTPases, and JNK, which may vary in different cell types, but commonly regulate cell polarity and movement via changes in cytoskeletal dynamics [6], [24]–[29].

Dsh is conserved in Bilateria and functions in dorsal patterning [11], [30], [31], cell polarity [15], [25], [32], [33], gastrulation movements [25], [27], [34], [35], and neural tube closure [26], [36]. In *X. laevis,* localization of Dsh to the presumptive dorsal side of the early embryo and subsequent nuclear localization of β-catenin during cleavage stages results in activation of several target genes, including *siamois, Xnr3,* and *Xbra* [13], [37]–[44]. Thereafter, Xbra induces expression of Wnt11, and downstream β-catenin-independent signaling through Dsh regulates convergent extension movements in the mesoderm and neural ectoderm involved in gastrulation and neural tube closure [45]–[47].

Dsh is a modular protein comprised of multiple conserved domains including the DIX, PDZ and DEP domains that have partially separable signaling functions [15], [48]–[50]. The DIX domain is an alpha helical motif located near the N-terminus that mediates homo- and hetero-dimerization between Dvl proteins [30], [51], [52], Axin proteins [51], [53], Ccd1/DIXDC1 proteins [51], [54], [55], plus interaction with at least one protein that lacks a DIX domain, Actin [56]. Evidence from different organisms has suggested that the DIX domain is required for Wnt/β-catenin signaling as opposed to β-catenin-independent forms of signaling. In *Drosophila melanogaster*, the DIX domain is necessary to increase β-catenin levels in the nucleus, but not to rescue PCP pathway defects [20], [48], [49]. In mammalian cells, the DIX domain is required to increase nuclear β-catenin and to activate Tcf/Lef-dependent transcription, but not to activate JNK activity [33], [50], [51], [55], [57]. Similarly, over-expression studies have confirmed that an *X. laevis* Dsh ortholog (Xdsh; NP_001084096) requires the DIX domain for activation of Wnt/β-catenin signaling [30], [33], [53], [56], but not to affect convergent extension movements, a hallmark of PCP pathway activity [25], [46], [58], [59].

To increase our understanding of the Dsh DIX domain and its protein partners we conducted a yeast 2-hybrid screen using the N-terminus of Dsh as bait. One Dsh-interacting protein isolated from this screen was the *X. laevis* ortholog of the transcriptional co-repressor, Homeodomain Interacting Protein Kinase-1 (XHIPK1/hipk1, EU980449, NP_001128533). Members of the Hipk1 protein family were first identified by virtue of binding to the homeobox genes Nkx1.2, NK-1, NK-3, Nkx2-5, and HoxD4 [60], and subsequently have been reported to also interact with p53, Daxx, and AML1 [60]–[63]. Hipk1 and the related Hipk2 are together required for neural tube closure, hematopoiesis, angiogenesis and vasculogenesis in mice [64]. Hipk2 but not Hipk1 has previously been implicated in Wnt/β-catenin signaling [65]–[67]. No Hipk family member has previously been shown to interact with Dsh, nor to influence β-catenin-independent forms of Wnt signaling.

Here, we characterize the functions of Hipk1 during *X. laevis* development in the context of the Wnt signaling pathways. The Hipk1 ortholog from *X. laevis* is expressed from the earliest stages of development and in a tissue-specific manner at later stages. Hipk1 binds to Dsh as shown by *in vitro* binding assays and co-immunoprecipitation from embryo extracts. Hipk1 also binds to the Lef/Tcf family member Tcf3. Hipk1 knock-down in the early embryo leads to a broadening of the expression domains of Wnt/β-catenin-responsive genes involved in dorsal specification, while knock-down in human tissue culture cells similarly indicates that Hipk1 can repress transcriptional activation of β-catenin-responsive promoter elements. Nevertheless, during gastrulation Hipk1 is necessary for transcriptional activation of a subset of Wnt-responsive genes in the involuting mesoderm, and disruption of Hipk1 by either over-expression or knock-down leads to severe defects in convergent extension cell movements. These data suggest that through interactions with both Dsh and Tcf3, Hipk1 acts as an important modulator of Wnt signaling during early vertebrate development.

## Results

### Hipk1 interacts with Dsh and Tcf3

To identify novel interactions regulating Dsh function, we performed a yeast-2-hybrid screen of an *X. laevis* unfertilized oocyte cDNA library, using the N-terminus of Dsh, including the DIX domain but not the PDZ or DEP domains, as bait. Among other potential interactors, we recovered three different clones encoding the Dsh DIX domain itself (Cheyette and Moon, data not shown), confirming prior reports that this domain mediates homodimerization [30], [51], [52] and validating our screen. A 954 nucleotide clone recovered in this screen, closely related to the 3′ region of the previously identified Hipk gene family, led to the focus of this study.

**Figure 1:**
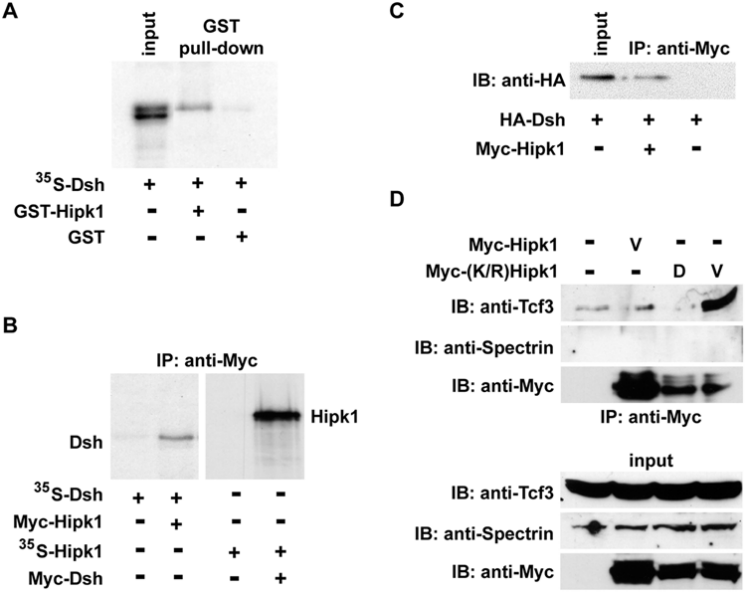
Hipk1 interacts with Dsh and Tcf3. (A) ^35^S-Met-labelled Dsh precipitates in a complex with the partial-length Hipk1 GST-tagged clone from the yeast-2-hybrid screen, but not with GST alone. (B) Immunoprecipitation (IP) of ^35^S-Met-labelled Dsh with Myc-tagged Hipk1 (left panel), and reciprocal ^35^S-Met-labelled Hipk1 IP with Myc-tagged Dsh (right panel), using in vitro translated proteins. (C) IP of Dsh with Hipk1 when both proteins are recombinantly expressed in *X. laevis* embryos. (D) Endogenous Tcf3 IP with recombinantly expressed Myc-(K/R) Hipk1 in ventral *X. laevis* embryos. An anti-Spectrin antibody was used as a negative control for the IP (top) and as a loading control for the input (bottom).

To confirm binding between Dsh and this putative partial-length Hipk clone, we tested whether an interaction could be recapitulated by in vitro pull-down assay. Supporting the yeast-2-hybrid results, ^35^S-labelled Dsh bound to a recombinantly expressed and purified polypeptide corresponding to this clone fused to GST, but not to GST alone (Fig. 1A second *vs.* third lane).

A corresponding full-length cDNA was isolated from *X. laevis* unfertilized egg and tadpole libraries (Materials and Methods). By sequence comparison this is the *X. laevis* ortholog of Hipk1 (Fig. S1, S2). Conserved domains found in this gene include a dual-specificity kinase domain (KD) that encodes both serine/threonine and tyrosine kinase consensus motifs. Within this kinase domain, an ATP-binding motif whose sequence diverges between Hipk paralogs corresponds precisely to the Hipk1 subfamily consensus (Fig. S1). Other conserved motifs include the homeodomain interaction domain (HID), a speckle retention sequence and overlapping PEST domain (SRS/PEST), and a C-terminal Tyr/His-rich (Y/H) region [60]. The clone originating from the yeast-2-hybrid prey library corresponds roughly to the C-terminal third of Hipk1 excluding the final 70 amino acids (codons 786–1104). We have provisionally labeled this the Dishevelled-Interacting Region (DIR) (Fig. S1, S2B). Except where otherwise noted, all experiments in this study involve manipulations of this *X. laevis* ortholog of Hipk1 and the corresponding mRNA and protein.

To test whether full-length Hipk1 and Dsh interact, we performed further co-immunoprecipitation experiments with in vitro translated tagged proteins. Using this methodology, ^35^S-Dsh bound specifically to Myc-Hipk1 (Fig. 1B left panel), and reciprocally ^35^S-Hipk1 bound specifically to Myc-Dsh (Fig. 1B right panel). We next tested whether this interaction occurs in *X. laevis* embryos and in cultured human cells. In extracts of *X. laevis* embryos that had been injected with synthetic RNAs, HA-Dsh co-immunoprecipitated with Myc-tagged Hipk1 (Fig. 1C). Similarly, in extracts from a human embryonic kidney cell line (HEK293T) that had been transfected with Dsh, affinity purification of Dsh followed by tandem mass spectrometry revealed that human HIPK1 associates with Dsh in this cell line (Angers, Maccoss and Moon, data not shown). These results show that Hipk1 and Dsh can exist as a complex *in vivo*, and strongly suggest that they are direct interactors.

A human Dsh protein, DVL3, forms a complex with Tcf4 on DNA [68]. As Hipk proteins have previously been shown to function as transcriptional co-repressors (Kim et al, 1998), it was logical to ask whether Hipk1 might also interact with Lef/Tcf proteins such as Tcf3 (P70062), an HMG-box repressor that operates as part of the Wnt/β-catenin pathway in *X. laevis* [40], [69]–[71]. We accordingly tested whether endogenous Tcf3 co-immunoprecipitates with Hipk1 from extracts of developing *X. laevis* embryos injected with synthetic RNA encoding Myc-Hipk1. Endogenous Tcf3 did not detectably co-immunoprecipitate with Myc-tagged Hipk1 (Fig. 1D second lane). Notwithstanding this initial negative result, we pursued this hypothesis further by asking whether interaction between these proteins might be regulated by activity of the Hipk1 kinase domain, and therefore exhibit a more stable configuration if this domain were inactivated. We used site-directed mutagenesis to create a lysine to arginine mutation at position 217 of *X. laevis* hipk1 ((K/R)Hipk1) within the conserved ATP-binding motif. By analogy to previous studies using mouse orthologs, this mutation is predicted to abolish kinase activity. Consistent with our hypothesis, Myc-(K/R)Hipk1 associated with endogenous Tcf3 (Fig. 1D fourth lane). Furthermore, this interaction was specific for the ventral, as opposed to the dorsal, side of the embryo (Fig. 1D fourth *vs.* third lane). This is of potential relevance to development because Tcf3 represses β-catenin-mediated transcription specifically on the ventral side of the embryo, even in the presence of low levels of nuclear β-catenin [42], [43], [69], [70].

### Hipk1 is expressed throughout development

**Figure 2:**
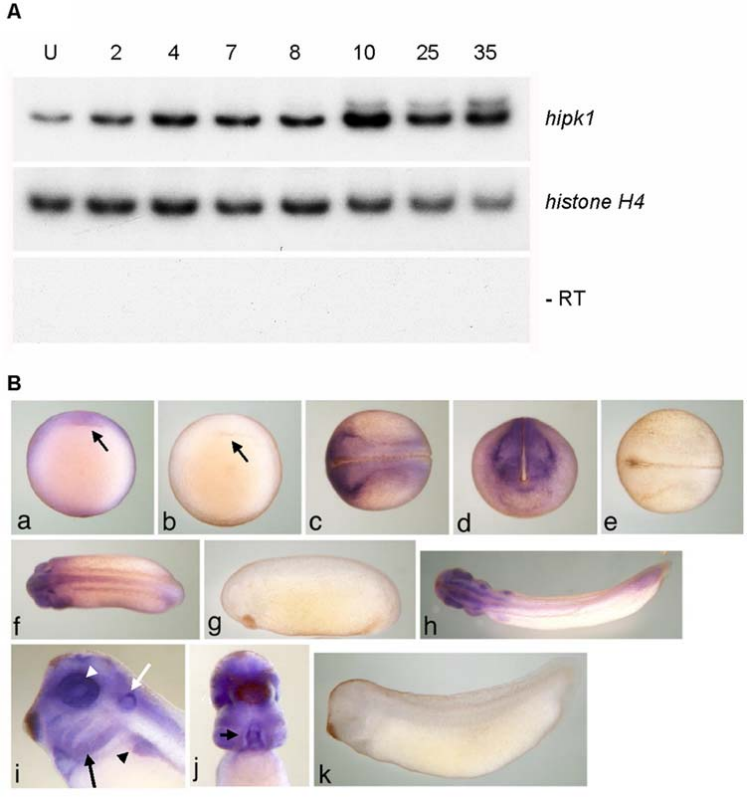
Hipk1 is expressed throughout vertebrate development. (A) Levels of the *X. laevis* hipk1 mRNA were measured by RT-PCR at the following developmental stages: Unfertilized egg (U), 2-cell (2), 4-cell (4), blastula (Stage 7, 8), gastrula (Stage 10), neurula (Stage 17), tail bud (Stage 25), and early tadpole (Stage 35). *Histone H4* was used as a loading control, and reactions without reverse transcriptase (−RT) performed to rule out genomic DNA contamination. (B) WISH of the hipk1 transcript during *X. laevis* development. At Stage 10 (a, ventral aspect) signal is detected in the marginal zone and animal pole with greater staining above the dorsal lip of the blastopore (arrow). At Stage 17 (c, dorsal aspect; d, anterior aspect) signal is apparent at the margins of the lateral and anterior neural plate. At Stage 25 (f) and Stage 35 (h), signal is present in dorsal anterior regions and in the tail bud. Close-up of the Stage 35 head (i, lateral aspect) reveals expression in the branchial arches (black arrow), otic vesicle especially dorsally (white arrow), pronephros (black arrowhead), and retina (white arrowhead). Close-up of the Stage 35 head/torso (j, ventral aspect) shows high expression in the heart (arrow). Sense controls (b, e, g, k).

We characterized temporal and spatial expression of the *X. laevis* ortholog using RT-PCR and whole-mount in situ hybridization (WISH). Temporally, Hipk1 is expressed throughout *X. laevis* embryonic development from the unfertilized egg (U) through early tadpole stages (Stage 35) (Fig. 2A). There is a relative peak in expression at the onset of gastrulation (Stage 10), at which time its spatial distribution is strongest in the marginal zone (Fig. 2A, 2B panel a). Later, in the neurula, it is present in the anterior and lateral neural plate and in the placodal anlagen (Fig. 2B panels c, d). Expression at the tail bud stage (Stage 25) occurs in the eye vesicle, presumptive heart anlage, ear placode, pronephric anlage, anterior CNS (brain and spinal cord), and tail blastema (Fig. 2B panel f). By early tadpole stages (Stage 35), it is concentrated in dorsal anterior structures and at the tip of the tail, as well as in the eye, heart, otic vesicle, pronephric system, branchial arches, CNS, and tail bud, but with little or no epidermal or endodermal expression (Fig. 2B panels h–j). The tissue distribution of Hipk1 in *X. laevis* at neurula stages and later is similar to that previously reported for Hipk family members in the mouse [62], [64]. Nonetheless, since expression earlier than neurula stages has not previously been reported, our data in *X. laevis* suggest a previously unsuspected function for Hipk1 during earlier stages of vertebrate embryogenesis.

### Hipk1 regulates Wnt/β-catenin target genes during dorsal specification and gastrulation

**Figure 3:**
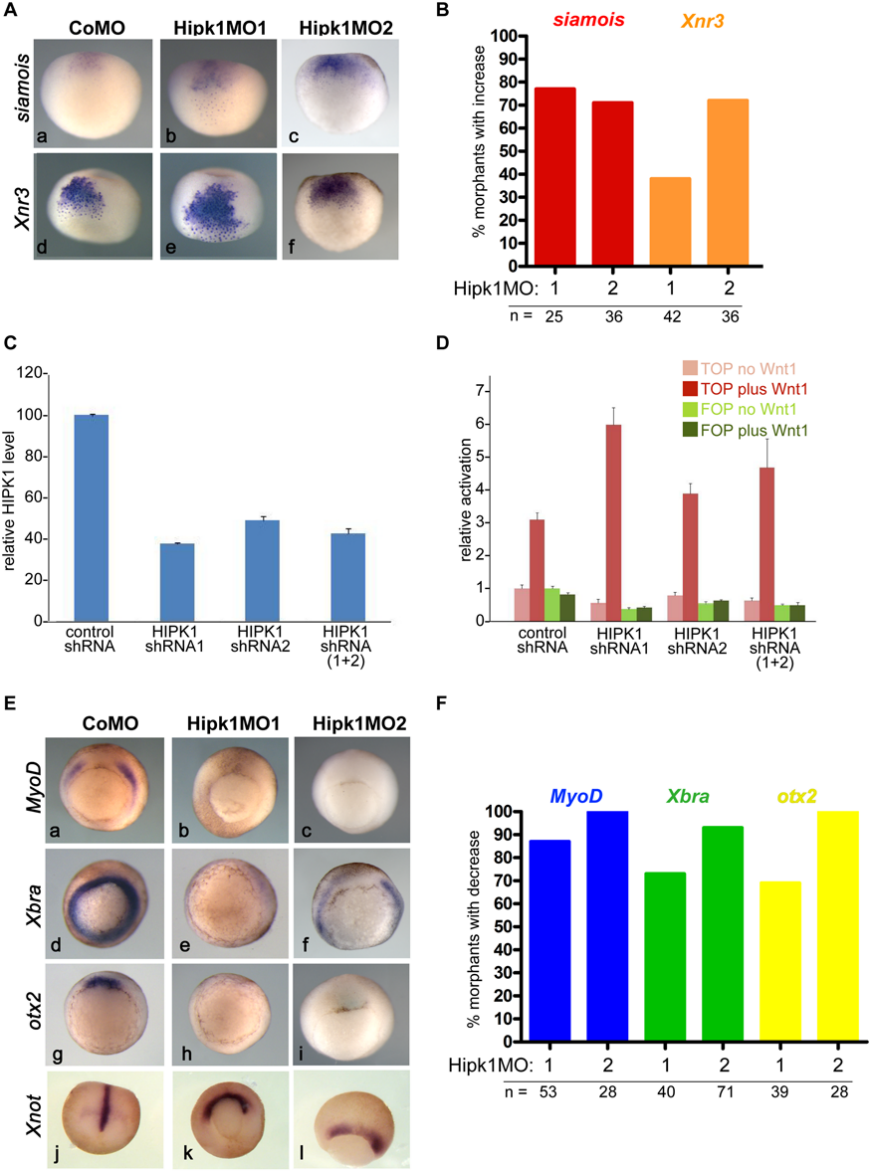
Hipk1 regulates Wnt/β-catenin targets in the early embryo. (A) Morpholinos against Hipk1 expand expression domains of the Wnt-responsive dorsal patterning genes *siamois* (a–c) and *Xnr3* (d–f). Embryos were injected with CoMO (40 ng per embryo), Hipk1MO1 (40 ng per embryo), or Hipk1MO2 (20 ng per embryo) in the DMZ of 2 dorsal blastomeres at the 4-cell stage. (B) Quantification of results in A. (C, D) ShRNA plasmids targeted against HIPK1 reduce HIPK1 levels in human embryonic kidney cells (C) and potentiate the response of a β-catenin reporter construct to co-transfected Wnt1 (D). (E) Morpholinos against Hipk1 eliminate or reduce expression of the Wnt-responsive genes *MyoD* (a–c) *Xbra* (d–f), and *otx2* (g–i), but do not similarly eliminate expression of *Xnot* (j–l), a marker for axial mesoderm. Embryos were injected as in A. (F) Quantification of results in E.

To determine whether Hipk1 modulates Wnt/β-catenin-dependent gene activation during early development, we used morpholino oligonucleotides to knock-down endogenous Hipk1 levels in *X. laevis* embryos. Two different Hipk1 morpholinos (Hipk1MO1, and Hipk1MO2; see Materials and Methods & Fig. S4) versus a control morpholino (CoMO) were injected into the dorsal marginal zone (DMZ) and injected embryos (morphants) cultured to late blastula and early gastrula stages for gene expression analysis. Using this technique the earliest differences we were able to detect were in the expression domains of *siamois* and *Xnr3*, Wnt/β-catenin-responsive genes whose activation reflects initial dorsal specification prior to the onset of gastrulation [38], [69], [72]–[75]. The expression domains of both *siamois* and *Xnr3* were expanded in Hipk1 morphants when compared to controls (Fig. 3A panels b, c and e, f compared to a and d respectively). This effect was consistent between morphants: more than 75% of embryos injected with 40 nanograms per embryo of Hipk1MO1 exhibited expanded *siamois* expression whereas more than 35% exhibited expanded *Xnr3* expression, and Hipk1MO2 had similar effects (Fig. 3B). The spatially expanded domains of these dorsal patterning genes in Hipk1 morphants supports the hypothesis, also suggested by our biochemical studies (Fig. 1D), that during early *X. laevis* development Hipk1 cooperates with Tcf3 to repress Wnt/β-catenin pathway target gene activation in ventral tissues [69], [70].

In order to confirm that endogenous Hipk1 can antagonize Wnt/β-catenin signaling in another system, we used siRNA strategies to knock-down the human HIPK1 ortholog in HEK293T cells, and then assessed Wnt/β-catenin signaling using a β-catenin-dependent reporter plasmid in which luciferase expression is driven by optimal Lef/Tcf promoters [76], [77]. To achieve HIPK1 mRNA knock-down, we transiently transfected tissue culture cells with plasmids expressing three different short hairpin RNAs (shRNAs): a control shRNA versus two experimental shRNAs targeted against different parts of the HIPK1 transcript. The ability of these plasmids, alone and in combination, to specifically knock down HIPK1 mRNA transcripts was validated by Quantitative Reverse Transcriptase PCR (QPCR): HIPK1 shRNA1 reduced HIPK1 mRNA levels to 37.5% of control, HIPK1 shRNA2 reduced levels to 49.0% of control, and a 1∶1 combination of HIPK1 shRNA1∶shRNA2 reduced levels to 39.0% of control (Fig. 3C). Each of these knock-down conditions potentiated the β-catenin reporter response to co-transfected Wnt1 (Fig. 3D). Moreover, the degree of Wnt1 potentiation inversely correlated with the levels of HIPK1 mRNA obtained under each condition (Fig. 3C vs. 3D). These findings were independently corroborated using an alternate siRNA methodology and reporter assay in a different cell line (Fig. S3A). Furthermore, over-expression of Hipk1 antagonized Wnt/β-catenin signaling read-outs in both human tissue culture cells and in *X. laevis* embryos (Fig. S3B, C). These data demonstrate that in HEK293T mammalian cells as well as in the early vertebrate embryo, Hipk1 can inhibit transcriptional activation mediated by the Wnt/β-catenin pathway.

The onset of *otx2* expression in Spemann's Organizer begins at gastrulation, at which time *MyoD* and *Xbra* expression in involuting mesoderm is activated by Wnt/β-catenin signaling [78]–[81]. Expression of all three of these genes was nearly abolished in Hipk1 morphants (Fig. 3E panels a–i). This result was highly reproducible: expression of *MyoD*, *Xbra*, and *otx2* was markedly diminished in at least 70% of embryos at doses of either 40 nanograms per embryo of Hipk1MO1 or 32 nanograms per embryo of Hipk1MO2 (Fig. 3F). The loss of these markers did not merely reflect a delay in gastrulation, because their expression did not recover when assessed at later time points in embryos that had progressed further developmentally (Louie, Cheyette and Moon, data not shown). Similarly, the reduction in these markers did not reflect a more general failure in tissue-specific gene induction, because *Xnot*, a signaling-responsive gene that is expressed in notochord mesoderm [82], was normal in intensity although altered in its pattern (Fig. 3E panels k, l vs. j). We interpret this data to indicate that in addition to inhibiting the Wnt/β-catenin pathway during dorsal-ventral axis specification, endogenous Hipk1 also significantly contributes to activation of some Wnt/β-catenin target genes in the mesoderm at the onset of gastrulation. Not unlike reports that the Dsh-associated protein Dact1 (Dapper/Frodo) can alternately activate or inhibit the Wnt/β-catenin pathway under different conditions [83]–[85], we have similarly found that in some cell lines Hipk1 can contribute to transcriptional activation (Conrad, Cheyette and Moon, data not shown), supporting the hypothesis that signaling functions of HIPK1 are context-dependent.

### Hipk1 is a nuclear protein that directly affects Wnt/β-catenin target gene activation

**Figure 4:**
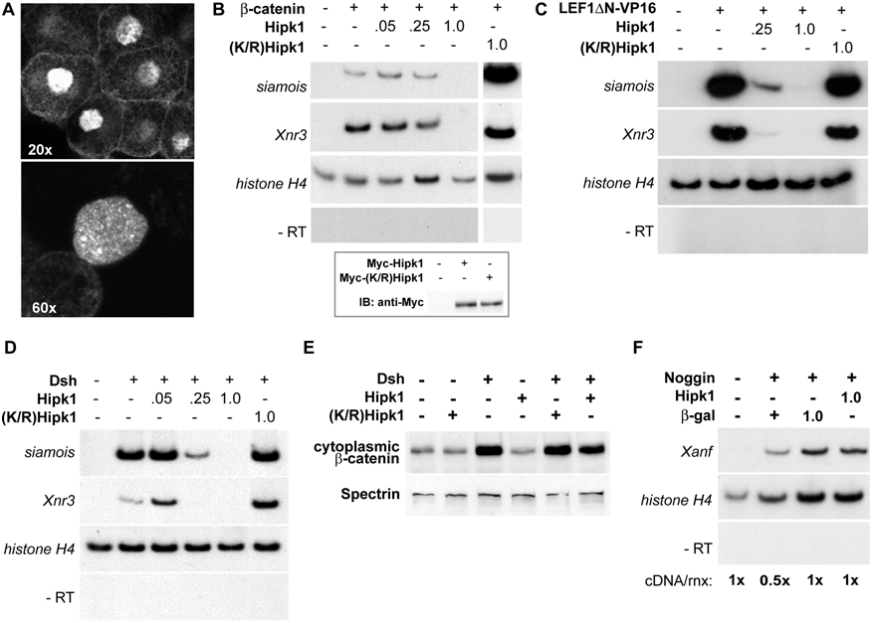
Hipk1 is nuclear and acts at the level of transcription during *X. laevis* development. (A) Confocal micrographs of animal pole explants recombinantly expressing Myc-tagged Hipk1 and visualized with anti-Myc antibody. Hipk1 localizes strongly to the nucleus and nuclear speckles, with low-levels of apparent extra-nuclear signal near the plasma membrane. (B–D) Hipk1 over-expression blocks activation of the Wnt-responsive genes *siamois* and *Xnr3* downstream of β-catenin (B), Lef/Tcf (C), and Dsh (D). *X. laevis* embryos were uninjected (lanes furthest on left in all panels) or injected with synthetic RNAs encoding β-catenin (B), LEF1ΔN-VP16 (C) or Dsh (D). Nanogram quantities of RNAs encoding Myc-Hipk1, Myc-(K/R)Hipk1 or β-galactosidase were co-injected as indicated. RT-PCR was used to assess target gene activation; *histone H4* was assessed as a loading control, and reactions without reverse transcriptase (−RT) performed to rule out genomic DNA contamination. (B, inset) Myc-tagged Hipk1 and (K/R)Hipk1, injected at 1 ng per blastomere, are expressed at comparable levels in *X. laevis* as demonstrated by western blot. (E) Over-expression of Dsh in HEK293T cells leads to an increase in cytoplasmic β-catenin that is not affected either positively or negatively by over-expression of Hipk1. (F) Induction of *Xanf* by Noggin in *X. laevis* animal caps is not affected by over-expression of Hipk1.

Previous studies of mammalian Hipk1 orthologs have shown that they are transcriptional regulators that localize to nuclear speckles when expressed in tissue culture cells [63], [64]. We tested whether the Hipk1 protein has a similar subcellular distribution in *X. laevis*, and confirmed that Myc-Hipk1 does indeed predominantly localize to the nucleus and to nuclear speckles in *X. laevis* animal cap explants (Fig. 4A). This suggests that Hipk1 is likely to act as a nuclear regulator of Wnt-dependent gene transcription during vertebrate development.

To test this hypothesis, we asked whether over-expression of Hipk1 in *X. laevis* embryos could alter activation of Wnt/β-catenin pathway target genes even in the presence of over-expressed β-catenin. Consistent with a direct role in transcriptional regulation, Hipk1 but not (K/R)Hipk1 inhibited induction of *Xnr3* and *siamois* in the presence of over-expressed β-catenin (Fig. 4B third, fourth and fifth *vs.* second and sixth lanes). Furthermore, an activated Lef/Tcf-viral transcription factor chimera, LEF1ΔN-VP16 [86], was also inhibited by Hipk1 but not by (K/R)Hipk1 (Fig. 4C third and fourth *vs.* second and fifth lanes). Finally, the ability of over-expressed Dsh to cause Wnt/β-catenin target gene activation was blocked by co-expressed Hipk1 but not (K/R)Hipk1 (Fig. 4D fourth and fifth *vs.* second and sixth lanes), even though cytoplasmic β-catenin levels were elevated by Dsh when Hipk1 was co-expressed in cultured cells (Fig. 4E sixth *vs.* third lane). In contrast to these findings for Wnt/β-catenin target genes, over-expression of Hipk1 did not alter transcriptional activation of the Noggin signaling target *Xanf1* [87] (Fig. 4F). Together these results suggest that Hipk1 exerts its effects on the Wnt/β-catenin pathway at the level of transcriptional regulation downstream of β-catenin in the nucleus. They further demonstrate that this effect on transcription is not a generalized phenomenon encompassing other signal pathway targets.

### Hipk1 gain- and loss-of-function cause similar gastrulation and neural tube closure phenotypes

**Figure 5:**
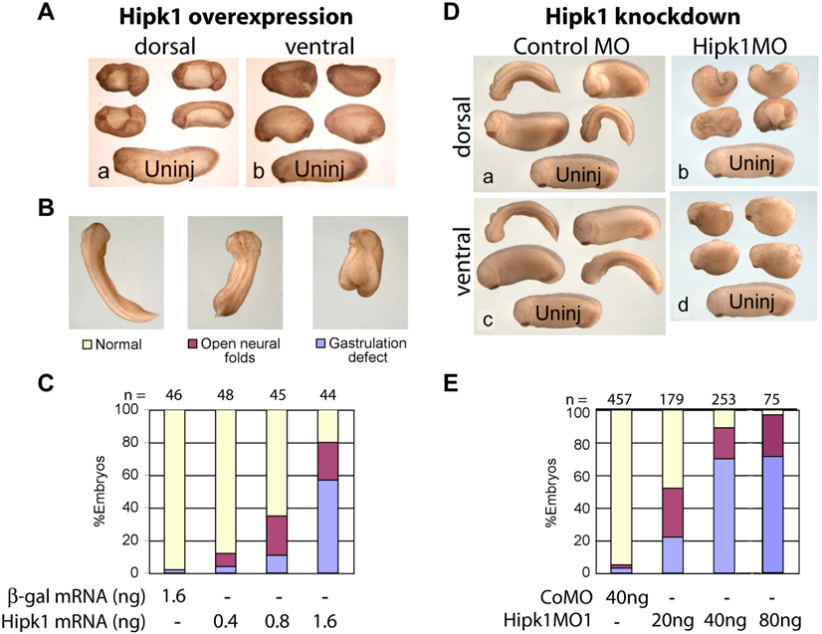
Hipk1 over-expression and knock-down both produce gastrulation and neural tube defects. (A) Over-expression of Hipk1 dorsally results in shortened embryos with gastrulation defects and an open neural tube both anteriorly and posteriorly (a). When Hipk1 is over-expressed ventrally, the neural tube closes, but embryos are shortened and fail to complete blastopore closure (b). Synthetic Hipk1 RNA was injected into the marginal zone of 2 dorsal or 2 ventral blastomeres at the 4-cell stage. An uninjected (Uninj) control embryo is shown at the bottom of each panel for comparison. (B) Representative phenotypes used for quantification in C and E. (C) Graphical representation of phenotype distribution resulting from increasing quantities of Hipk1 RNA injected into the DMZ. (D) Injection of Hipk1MO results in gastrulation defects and an open neural tube similar to the over-expression phenotypes in A–C. CoMO (a, c) or Hipk1MO (b, d) was injected either dorsally (a, b), or ventrally (c, d). Embryos injected with the control morpholino did not differ from uninjected embryos (Uninj) shown at the bottom of each panel. (E) Hipk1MO produces gastrulation defects in a dose-dependent manner. Anterior is to the left in A, D, and at the top in B.

The striking effects of Hipk1 knock-down on some mesoderm markers suggested that manipulating Hipk1 might also cause severe disruptions in the cell movements that accompany specification of these tissues during gastrulation. To test whether Hipk1 plays a role in regulating these and other cell movements, we performed over-expression and loss-of-function phenotypic experiments in whole embryos. Injection of synthetic RNA encoding Hipk1 into the DMZ resulted in severe gastrulation and neural tube closure defects, demonstrated by a failure to close the blastopore and to fuse the neural tube (Fig. 5A panel a). The percentage of dorsally-injected embryos with the severe gastrulation phenotype was dose-dependent, with higher doses producing more severe effects (Fig. 5B–C, Supplemental Table S1). In contrast, injection of Hipk1 RNA into the ventral marginal zone (VMZ) resulted in less severely affected embryos with a shortened anterior-posterior length, but a nearly closed blastopore and normal neural tube (Fig. 5A panel b).

One feature of molecules involved in β-catenin-independent pathways, particularly the PCP pathway, is that over-expression phenotypes resemble loss-of-function phenotypes at both the cellular and embryonic level [88], [89]. Consistent with a role in such a pathway, phenotypes in Hipk1 morphants closely resembled those observed in embryos over-expressing Hipk1, including comparisons between dorsal versus ventral injections. When either Hipk1MO was injected into the DMZ, phenotypes included shortened embryos with defects in blastopore closure and in neural tube closure (Fig. 5D panel b; 5E). When either Hipk1MO was injected into the VMZ, phenotypes included shortened embryos with defects in blastopore closure but with a normal neural tube (Fig. 5D panel d). In both cases, control morphants were indistinguishable from uninjected embryos (Fig. 5D panels a, c; 5E). Effects of Hipk1MOs were dose-dependent: 20 nanograms of Hipk1MO1 per embryo injected into the DMZ affected 52% (n = 179), 40 nanograms per embryo affected 89% (n = 253), and 80 nanograms per embryo affected 97% (n = 75) (Supplemental Table S2). Hipk1MO2 caused identical phenotypes to Hipk1MO1 at doses between 8–32 nanograms per embryo; higher doses led to significant lethality (Supplemental Table S2 and data not shown). This is consistent with molecular observations that Hipk1MO2 is less specific than Hipk1MO1 at high doses (Fig. S4). Concordant with the penetrance of its effects on gene activation during gastrulation (Fig. 3F), 70% of Hipk1 morphants injected with 40 nanograms of Hipk1MO1 per embryo in the DMZ exhibited the severe gastrulation phenotype (Fig. 5E third bar; Supplemental Table S2). To summarize, phenotypic data from both Hipk1 over-expression and knock-down suggest that these molecular manipulations lead to substantial defects in the cell movements necessary for gastrulation and neural tube closure, consistent with either a direct or upstream regulatory role for Hipk1 in a β-catenin-independent pathway, such as PCP.

### Hipk1 morphants exhibit delayed gastrulation and impaired convergent extension movements

**Figure 6:**
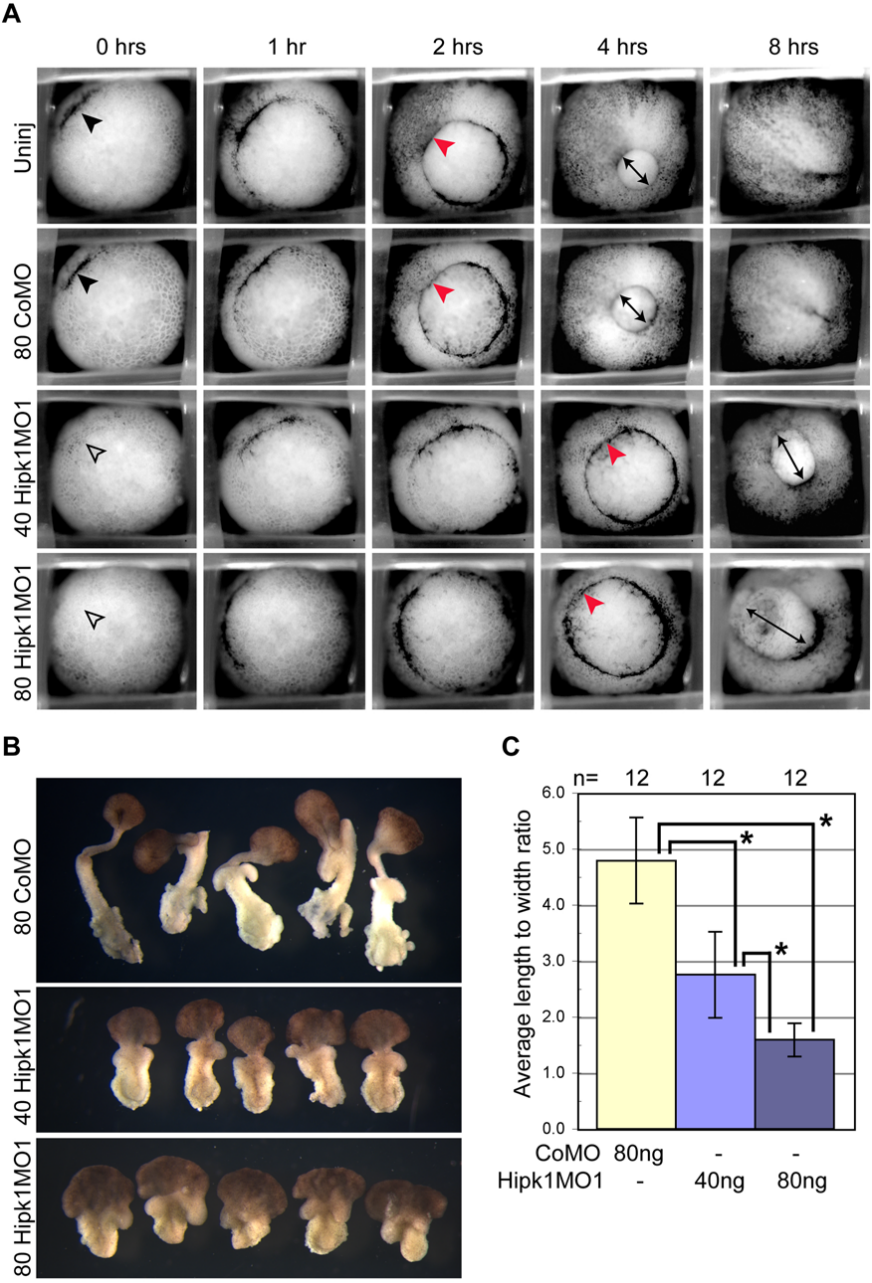
Hipk1 is required for gastrulation and convergent extension movements. Morpholino-mediated knock-down of Hipk1 perturbs initiation and progression of gastrulation. (A) Hipk1 morphant embryos exhibit delay of dorsal blastopore lip formation and do not close the blastopore compared to controls (see text). Frames from movies of gastrulating embryos in parallel are presented, from embryos that were either uninjected (Uninj) or injected in the DMZ at the 4-cell stage with 80 ng of CoMo, 40 ng of Hipk1MO1, or 80 ng of Hipk1MO1 as labeled. Elapsed time after appearance of the dorsal lip (black arrowhead) in controls (0 hrs) is indicated. See text for details. (B and C) Keller explant sandwiches from Hipk1 morphant embryos exhibit dose-dependent failure of convergent extension movements compared to controls. DMZ explants were cultured as sandwiches to Stage 19 and average LWR determined. LWR was significantly decreased in Hipk1 morphants compared to controls, and this was dose-dependent (*Pr(T>t) = 0.00001 using 2-sample t test with equal variances).

Since both over-expression and knock-down phenotypes at neurula stages are indicative of gastrulation failure, we collected time-lapse movies of Hipk1 morphant embryos and control embryos in parallel, in order to determine the time course of this developmental phenotype. Uninjected and control morpholino-injected embryos simultaneously reached Stage 10+, characterized by initial formation of the dorsal blastopore lip (Fig. 6A black arrowheads in upper two rows); this time-point was set to 0 hours for all conditions. At 2 hours, control embryos reached Stage 11, marked by formation of a fully circular blastopore ring and initial involution of dorsal mesoderm (Fig. 6A red arrowheads in upper two rows). At 4 hours, the blastopore ring in controls had constricted to a circle less than half its initial diameter (Fig. 6A double-headed black arrows in upper two rows) reflective of Stage 12. By 8 hours, control embryos had completed blastopore closure and had begun to extend an anterior-posterior (AP) axis (Stage 13; Fig. 6A right-most panels in upper two rows).

Compared to control embryos, the first appearance of the blastopore lip was delayed by an average of 18 minutes in Hipk1 morphants injected with 40 nanograms of Hipk1MO1, and by 30 minutes in Hipk1 morphants injected with 80 nanograms of Hipk1MO1 (Fig. 6A open arrowheads in lower two rows). Involution of mesoderm was delayed by more than 2 hours in Hipk1 morphants, and was finally apparent at 4 hours (Fig. 6A red arrows in lower two rows). Constriction of the blastopore ring was delayed by a similar interval (Fig. 6A double-headed black arrows in lower two rows). By 8 hours, most Hipk1 morphants still had an open blastopore and had not yet begun to extend an AP axis (Fig. 6A right-most panels in lower two rows). In the end, greater than 75% of Hipk1 morphants exhibited a shortened AP axis and failed to completely close the blastopore.

To test whether this Hipk1 morphant phenotype could be explained by impaired convergent extension movements, Keller explant sandwiches were prepared from control and Hipk1 morphant embryos [90]. DMZ explants were removed from Stage 10 control or Hipk1 morphant embryos and explant sandwiches were cultured until Stage 19 (Fig. 6B; S5) when mean length-to-width ratio (LWR) was calculated (Materials and Methods). Mean LWR was significantly reduced in Hipk1 morphant embryos compared to controls, and this was sensitive to the dose of Hipk1MO injected (Fig. 6C; high dose Hipk1MO1 LWR = 1.6; low dose Hipk1MO1 LWR = 2.8; CoMO LWR = 4.8). Interestingly, both the ectoderm and mesoderm of Hipk1 morphant explant sandwiches failed to elongate, demonstrating that loss of Hipk1 leads to convergent extension defects in both of these germ layers.

### Hipk1 and Dsh act together in gastrulation and neural tube closure

**Figure 7:**
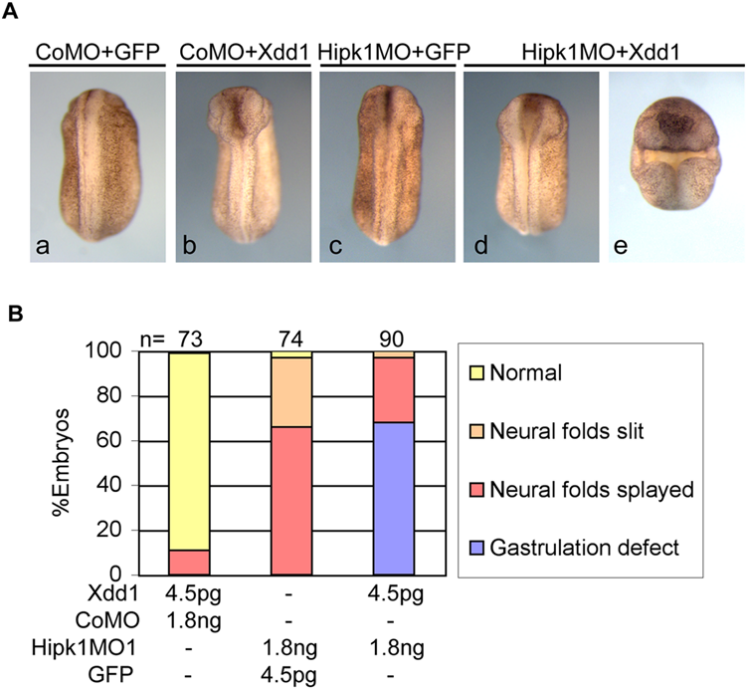
Hipk1 knock-down and Xdd1 over-expression synergize to perturb gastrulation. (A) Reduction of Hipk1 levels by morpholino, or interference with Dsh function by expression of a dominant negative protein (Xdd1), each produce no gastrulation defects at low doses, with only mild neural tube closure defects (b, c *vs.* control a). Combining these low-dose treatments produces gastrulation defects plus more severe neural tube closure defects (d, e). (B) Gastrulation defects are present in 68% of embryos in the Xdd1+Hipk1MO group, but not at all in groups injected with either Xdd1 RNA or Hipk1MO alone.

Xdd1 is a mutant form of the *X. laevis* Dsh ortholog that has been shown in numerous studies to have dominant-negative effects on both β-catenin-dependent and β-catenin-independent signaling read-outs including: Wnt8-induced dorsal-ventral axis duplication [34], *siamois* induction [91], blastopore closure [92], and convergent extension movements [34]. We noticed that phenotypes produced by Hipk1 over-expression and knock-down very closely resemble phenotypes resulting from Xdd1 over-expression. To determine whether cooperation between Hipk1 and Dsh is required during development, we co-injected an Xdd1 synthetic RNA and Hipk1MO1 into the DMZ of 4-cell embryos. Doses of Xdd1 RNA and the Hipk1MO were titrated so that no gastrulation defects were observed when either of these reagents was injected with control reagents (GFP RNA or CoMO) (Fig. 7A panels b–c). Both reagents were then co-injected at these doses into the marginal zone of the two dorsal blastomeres at the 4-cell stage. Severe gastrulation defects were observed in 68% of embryos co-injected with Xdd1 and Hipk1MO, whereas no embryos co-injected with either reagent plus control displayed abnormal gastrulation (Fig. 7A panel e *vs.* b, c; 7B). These data support the conclusion that Dsh and Hipk1 cooperatively regulate molecular and cellular events underlying gastrulation and neural tube closure.

## Discussion

Dsh is a critical signaling molecule involved in many Wnt-related activities during gastrulation including cell fate determination, cell shape, and cell movement. Although Dsh is expressed fairly ubiquitously, it exhibits variable intracellular localization, signaling activities, and protein interactions over the course of early *X. laevis* development [14], [15], [93]. Consequently, it is important to identify binding partners of Dsh that mediate alternate developmental functions. In this study we have identified one such protein as the nuclear kinase Hipk1, and have shown that it also can interact with the Wnt/β-catenin transcriptional co-repressor Tcf3.

Proper germ layer specification reflected by induction of genes such as *MyoD, Xbra,* and *otx2* is required to promote cell movements necessary for gastrulation [58]. The combined disruptions in gene expression and cell movements exhibited by Hipk1 morphants are consistent with a role in activating β-catenin-dependent target genes in the involuting mesoderm, followed by effects on β-catenin-independent events in these tissues. Gain- and loss-of-function of several molecules involved in a β-catenin independent pathway produce the same or very similar convergent extension phenotypes, including Wnt11 [46], Lrp6 [91], Fz7 [44], [94]–[97], Stbm/Vang [98], [99], and PKCδ [100]. In contrast to Hipk1, these other genes are not required for induction of Wnt/β-catenin target genes such as *MyoD*, *Xbra*, or *otx2*. Although the strong phenotypic effects we observe on gastrulation, neural tube closure, and Keller explants could reflect direct participation of Hipk1 in a β-catenin-independent pathway such as PCP, the most parsimonious model is that Hipk1 functions upstream of these processes in Wnt/β-catenin transcriptional activation during gastrulation. This is consistent with our time-lapse analysis of gastrulation movements in Hipk1 morphant embryos, which documents changes as early as Stage 10 when specification of the mesoderm occurs. In future studies it will be interesting to examine whether Hipk1 helps regulate transcription of PCP pathway component genes, and even whether it interacts with PCP proteins such as Dsh in a cytoplasmic compartment, beyond its nuclear role in regulating transcription.

We have also observed in *X. laevis* embryos and in mammalian cells that Hipk1 can antagonize Wnt/β-catenin-mediated gene activation. Previous studies have characterized members of the Hipk protein family as transcriptional co-repressors [60], [63]. This, combined with the biochemical interaction of Hipk1 with Tcf3 and the broadened expression domains of dorsal patterning genes in Hipk1 morphants, suggests that Hipk1 and Tcf3 may cooperatively restrict activation of certain Wnt/β-catenin pathway targets to a circumscribed region of the dorsal embryo. It is interesting that effects of Hipk1 on the Wnt/β-catenin pathway depend on an intact kinase domain, and that this kinase activity also appears to negatively regulate the strength of this Hipk1/Tcf3 interaction. This suggests that cooperative functions of Hipk1 and Tcf3 may be regulated by the Hipk1 kinase. In this case Tcf3 or another factor in the same transcriptional complex such as Groucho [101], [102] might be a Hipk1 kinase substrate. One implication is that the expression of some developmentally-important genes may be coordinated by pathways that regulate Hipk1 kinase activity in conjunction with Wnt/β-catenin signaling.

It is less clear from our present study how Dsh may be mechanistically involved in the gene-regulatory functions of Hipk1. Although Dsh proteins are considered to be predominantly cytoplasmic, they also can localize to the nucleus and have been reported to interact with c-Jun and Tcf proteins [68], [103], [104]. As Hipk1 is predominantly nuclear and as it regulates gene transcription, we speculate that it may interact with a nuclear complex containing both Dsh and Lef/Tcf proteins.

In summary, we have characterized a role for Hipk1 in Wnt signaling during early embryonic development. Our data suggest that Hipk1 contributes to the changing functions of signaling through Dsh and Lef/Tcf proteins at the onset of gastrulation: from regulating genes involved in dorsal-ventral axis formation, to regulating genes involved in germ layer specification and subsequent cell movements.

## Materials and Methods

### Use of animals

Research protocols were approved by the Institutional Animal Care and Use Committees (IACUC) at the University of Washington and at the University of California, San Francisco.

### Isolation of X. laevis hipk1

The MATCHMAKER™ Two Hybrid System (CLONTECH) was used with an *X. laevis* unfertilized oocyte cDNA library. The fragment used as bait corresponds to amino acid residues 1–268 of Xdsh, encoding the N-terminus including the DIX domain but excluding the PDZ and DEP domains. Sequence obtained from the prey fragment obtained by the yeast-2-hybrid screen was used in 5′ and 3′ RACE to clone a full-length cDNA from *X. laevis* unfertilized egg and tadpole cDNA libraries (created by Jeff Brown using the Clontech Marathon RACE cDNA amplification kit). The full-length cDNA corresponding to *X. laevis* hipk1 was subcloned by standard techniques into pCS2+ and pCS2+MT (David Turner, FHCRC).

An amino acid substitution in the putative ATP-binding site of *X. laevis* hipk1 corresponding to Lys217Arg was created by SOE-PCR [105] using primers XHIPK1 K217R FP 5′-GATTGTAGCCATTAGAATTTTAAAGAATCAC-3′ and XHIPK1 K217R RP 5′-GTGAATCTTTAAAATTCTAATGGCTACAATC-3′. All constructs were verified by DNA sequencing.

### Phylogenetic analysis and sequence comparisons

A neighbor-joining phylogram was constructed using ClustalW 1.83 with the Gonnet matrix at the Genebee web-server (http://www.genebee.msu.su/clustal/clustal.php) and the following sequences: Human HIPK1 (NM_198268), human HIPK2 (NM_001113239.1), human HIPK3 (NP_005725), human HIPK4 (Q8NE63), mouse Hipk1 (NM_010432.2), mouse Hipk2 (NM_010433), mouse Hipk3 (NM_010434), mouse Hipk4 (Q3V016), chicken HIPK1 (XP_418007), chicken HIPK2 (XP_416335), chicken HIPK3 (ENSGALT00000019117), *X. tropicalis* hipk1 (NM_001082416), *X. tropicalis* hipk2 (NM_001079282.1), *X. tropicalis* hipk3 (NM_001079256.1), and *X. laevis* hipk1 (EU980449, identified in this study). Similar results were obtained using the Blosum62 matrix at Multalin (http://bioinfo.genopole-toulouse.prd.fr/multalin/multalin.html). A subset of these sequences was used to create the alignment in Figure S1 using Vector NTI software (Invitrogen).

### Protein interactions

The GST-Hipk1 fusion protein expression vector was constructed by in-frame subcloning of the *X. laevis* hipk1 partial-length yeast two-hybrid clone into pGEX (Pharmacia). GST-Hipk1 fusion protein was bound on Glutathione Sepharose 4B beads (Pharmacia 17-0756-01) according to standard techniques. Beads were washed 3 times in cold PBS, then 2 times in cold IP buffer (20 mM Tris pH 7.5, 150 mM NaCl, 0.1% Tween-20, 5 mM CaCl_2_). Xdsh was synthesized and labeled with ^35^S in vitro using the TNT® Quick Coupled Transcription/Translation Systems kit (Promega, Madison, WI, USA). 10 µl of ^35^S-Dsh was added to 25 µl of a 50% bead slurry, then rotated for 2 hours at 4°C, and then washed 4x in cold IP buffer. Half of the IP product was separated on a 10% SDS-PAGE gel and visualized by autoradiography. In Figure 1B, radiolabeled and unlabelled TNT reactions were mixed, then combined with Protein A Sepharose beads cross-linked with the 9e10 anti-Myc antibody. Immunoprecipitation and detection was conducted as described above.

For in vivo protein immunoprecipitations, approximately 0.7 ng of synthetic Myc-Hipk1 RNA was injected into each blastomere at the 2- to 4-cell stage and cultured in 0.1x MBS until stage 10. Embryos were lysed in 10 µl buffer (150 mM NaCl, 25 mM Tris pH 7.5, 1% Triton, and Roche Complete™ protease inhibitor added at the time of use) per embryo, incubated on ice for 15 minutes, then cleared by centrifugation. 10% of the supernatant was kept for input lanes, and the rest rotated with 9e10 anti-Myc monoclonal antibody-conjugated Protein A Sepharose beads overnight at 4°C. Beads were washed 4 times in cold IP buffer and products separated on a 7.5% SDS-PAGE gel. Detection of Dsh-HA or Tcf3 was by western blot using commercial primary antibodies, HRP-conjugated secondary antibodies, and the ECL detection system (Amersham Biosciences).

### Embryo manipulations

Wild-type *X. laevis* embryos were obtained, cultured, dissected, and analyzed according to standard protocols [106]. Probe synthesis and WISH were performed essentially as described [107] and in the following references: *Xtcf3* [71], *Xdsh* [11], *Xdd1* [34], *Xbra* [108], *Xnr3* (lab of Jim Smith), *siamois* [109], *MyoD* (Ralph Rupp), *Xnot* (David Kimelman), *pax6* and *otx2* (Grainger lab), *Krox20* [110].

Immunocytochemistry was performed on Stage 9 animal caps as previously described [111] using the 9e10 anti-Myc antibody at 1∶10 in 10% fetal bovine serum and PBST rotating overnight at 4°C, and the cy3 secondary antibody at 1∶750 rotating for 3 hours at room temperature. Animal caps were washed 3 times in PBST and mounted in Vectashield. Micrographs of *X. laevis* animal cap explants were collected as described [74] using a laser scanning confocal microscope (Nikon PCM 2000).

Keller explant sandwiches were prepared at Stage 10 as described [112] with any involuted mesoderm scraped off. Explant sandwiches were cultured in Danilchick's modified medium pH 8.3 (53 mM NaCl, 5 mM Na_2_CO_3_, 4.5 mM K-Gluconate, 32 mM Na-Gluconate, 1 mM CaCl_2_, 1 mM MgSO_4_, 0.1% BSA, and 50 µg/ml gentamycin sulfate) [113] under glass cover slips at 16°C until sibling embryos reached Stage 20.

Time-lapse video microscopy of whole embryos was done in the Keller lab as described [114]. Sibling embryos were either uninjected, injected with a high dose of control morpholino, or with Hipk1MO1. Three to five embryos for each treatment were immobilized in a monofilament nylon mesh while images were recorded from the vegetal side for approximately 12 hours. Image processing was accomplished using Metamorph software (Universal Imaging). Frames were extracted using NIH Image J, and edited in Adobe Photoshop.

### Synthetic RNAs and morpholinos

Synthetic RNA was transcribed in vitro from templates linearized with NotI, using the Ambion SP6 mMessage mMachine kit (Austin, TX, USA) and purified using G-50 columns (Princeton Separations). Morpholinos specific for *X. laevis* hipk1 were obtained from Gene Tools, LLC (Philomath, OR, USA) and were targeted against nucleotides in the 5′UTR and start codon: Hipk1MO1 (−23 to +1) TACCGCTGGTGTGTTGGGAAGATCA; Hipk1MO2 (−52 to −28) GCTGGTGAGAGGAGCTGCCTGGGAC (Fig. S4A). Morpholinos were tested for the ability to specifically diminish *X. laevis* hipk1 by co-translation experiments using an in vitro reticulocyte lysate transcription and translation assay (Invitrogen). Hipk1MO1 specifically antagonized the translation of *X. laevis* hipk1 compared to Xdsh. Hipk1MO2 did so also, although with less specificity at high doses (Fig. S4B–C). We therefore used Hipk1MO1 as our primary reagent, confirming results with low doses of Hipk1MO2 as described.

### RT-PCR

RT-PCR was carried out as described [115] with the following changes: cDNA was prepared using the Thermoscript RT-PCR System (Gibco/BRL), 50% of the mRNA was used for cDNA synthesis, and 1 µl of cDNA was used in a 25 µl PCR with 0.1 unit of Taq polymerase (Promega). Dilutions of cDNA template in the PCRs demonstrated that reactions were stopped in the linear phase of amplification after: 20 cycles (histone H4), 25–27 cycles (*Xnr3*), and 28–30 cycles (*siamois*) (not shown). Primers used for *X. laevis* hipk1 developmental RT-PCR were: 5′-CCGTGAATCAGATCAAGAGTCC-3′ (FP), 5′-GACGAACTACATGAGCCACTC-3′ (RP), and 27 cycles were performed, annealing at 60°C. Other primers as described elsewhere: *histone H4* [115], *Xnr3* and *siamois* [74], *otx2* and *Xanf2* [87].

### Luciferase reporter assay in Hipk1 knock-down cells

#### shRNA plasmid methodology/293T cells

Hairpin siRNA templates were designed, synthesized, and cloned into the pSilencer™ 4.1-CMV puro plasmid (Ambion), and confirmed by sequencing. The target of HIPK1 shRNA1 is GACACCUGAAGAACAUGAA, corresponding to nucleotides 1320 to 1338 of the HIPK1 ORF. The target of HIPK1 shRNA2 is GGCUUGCCAGCUGAAUAUC, corresponding to nucleotides 1225 to 1243 of the HIPK1 ORF. The control shRNA plasmid s encodes an siRNA with no targets in the mouse, human, or rat genome databases (Ambion). HEK293T cells were cultured in DMEM (High glucose) in 24-well plates and transfected using Lipofectamine 2000 (Invitrogen). Cells were transfected once with 800 ng shRNA plasmid(s) as indicated, cultured for 24 hours, then transfected again with 600 ng of the same shRNA(s) plus 170 ng pSuper(8x)TOPFlash or pSuper(8x)FOPFlash [89], 17 ng pRL-TK, +/−10 ng of plasmid encoding mouse Wnt1 (mWnt1-RSV). Cells were lysed and luciferase activities measured 48 hours after the second transfection using the Dual-Luciferase Reporter System (Promega) in a plate-reading luminometer (Luminoskan, Thermo Electric Corp). Relative firefly *vs.* renilla luciferase activity was calculated for each sample. Each shRNA transfection condition was performed and measured in quadruplicate cultures.

Efficacy of HIPK1 mRNA knock-down at the same time-point was assessed by QPCR essentially as described [116] using human cyclophilin (PPIA) mRNA levels as the internal standard. Two pairs of primers were used with comparable results: pair 1 = CAGTTTGCCACCCAATCCT (forward)+CAGCGGGTATCCAGTGTAAATT (reverse) amplifying nucleotides 3397–3462 of the HIPK1 ORF; pair 2 = CCCCCGTGGACCCTAAGT (forward)+CAATGTTGGCAGGGAAACTG (reverse) amplifying nucleotides 3167–3262 of the HIPK1 3′UTR.

#### Synthetic siRNA methodology/TREX 293 cells

A stable TREX 293 cell line expressing the tet repressor and human WNT1 under the control of a tet responsive promoter was cultured as described above and transduced with recombinant virus coding for a (12X)TOPFlash luciferase reporter and a constitutive renilla luciferase. Cells were seeded into a 96 well plate and transfected the following day using siPORT amine reagent (Ambion) with 100nM of a synthetic siRNA (Ambion) directed against the target sequence GGTCTAATGTCATCAGTTA, corresponding to nucleotides 2654–2672 of HIPK1. WNT1 expression was induced in the indicated wells by addition of tetracycline to the culture medium at a dose of 1 µg/ml, 15 hours before the luciferase assay was performed. 48 hours after transfection firefly and renilla luciferase activity were measured using the Dual-Luciferase Reporter System (Promega) and a Berthold Mithras plate-reading luminometer, essentially as described above.

Results were validated using two additional siRNAs: GCTCAATACAGTGCACAAT and GCCTCTGAATGTTGGTGTT, targeted against HIPK1 ORF nucleotides 1734–1792 and 2334–2352 respectively. By Q-RT-PCR these siRNAs reduced endogenous HIPK1 levels to 24% and 15% of control cells respectively; they also could potentiate TOPFlash reporter responsiveness to tetracycline-induced human WNT1 in TREX 293 cells (data not shown).

### Luciferase reporter assay in Hipk1 over-expressing cells

HEK293T cells were cultured and transfected as described above with 20 ng pTOPFlash luciferase reporter [76], 2 ng pRL-TK (renilla luciferase control plasmid), and expression constructs for the *X. laevis* Hipk1 and β-catenin gene products as shown. CS2P+ vector DNA was used to normalize all transfection mixes to 250 ng total DNA. Cells were cultured for 24 hours, then luciferase activity measured using the Dual-Luciferase Reporter System (Promega) as described above.

### β-catenin western blots

HEK293T cells were transfected in 10 cm plates with 7.5 µg DNA of each construct tested. CS2+ DNA was used to normalize total DNA in each transfection to 15 µg. Cells were lysed in 1.5 ml lysis buffer (50 mM Tris pH 7.5, 5 mM EDTA, 300 mM NaCl, 150 mM KCl, 1 mM DTT, 1% Nonidet P40, and Roche Complete™ protease inhibitor added at the time of use) at 4°C. 200 µl of whole cell lysate was treated with 40 µl Concanavalin A beads (Amersham) as previously described [47], and rotated for 4 hours at 4°C to remove membrane associated β-catenin. The supernatant was boiled in SDS sample buffer and loaded on a 7.5% SDS-PAGE gel. Blots were probed with anti-β-catenin at a concentration of 1∶1000 in 5% BSA in TBST and incubated overnight at 4°C, then followed with an anti-rabbit-HRP antibody at 1∶4000 in 5% milk in TBST incubated at room temperature for 1 hour.

## Supporting Information

Figure S1 Multiple sequence alignment of Hipk-family members and X. laevis hipk1. Residues identical to the X. laevis protein are shaded gray, and introduced gaps represented by a dash (-). Conserved domains defined in the literature are boxed and labeled. Sequence corresponding to the partial-length clone recovered in the yeast 2-hybrid screen is labeled as the “Dishevelled Interacting Region” (DIR). The conserved lysine in the ATP-binding portion of the kinase domain, which was mutated to create (K/R)Hipk1, is indicated in red. Accession numbers are listed in the Materials and Methods.
(0.08 MB PDF)

Figure S2 An X. laevis ortholog of Hipk1. (A) The X. laevis cDNA identified in this study is most similar to Hipk1 orthologs from other species, as opposed to other members of the Hipk gene family such as Hipk2, Hipk3, or Hipk4. (B) The primary sequence of X. laevis hipk1 is 1173 amino acids long and contains elements conserved in other family members including: a kinase domain (KD) with both Serine/Threonine and Tyrosine kinase consensus motifs, a homeodomain interaction domain (HID), a speckle retention sequence (SRS) and overlapping PEST domain, and a tyrosine-/histidine-rich (YH) domain. The extent of the clone from the yeast-2-hybrid screen is designated as the DIR (Dsh Interacting Region), corresponding to residues 786–1104.
(0.66 MB TIF)

Figure S3 Knock-down of HIPK1 potentiates WNT1 activity in a tetracycline-inducible cell line, whereas over-expression of Hipk1 antagonizes Wnt/β-catenin signaling. (A) SiRNA directed against human HIPK1 potentiates the response of a β-catenin-responsive reporter to human Wnt1 expressed from a tetracycline-inducible transgene (See also Materials and Methods). (B) Transfection of HEK293 cells with plasmid encoding the X. laevis Hipk1 ortholog inhibits activation of a β-catenin-responsive reporter (TOPFlash) by β-catenin in a dose-responsive manner. (C) X. laevis axis duplication assay. Co-injection of Hipk1, but not (K/R)Hipk1 or GFP synthetic RNAs, interferes with duplication of the embryonic axis induced by Wnt8.
(0.94 MB TIF)

Figure S4 Hipk1 morpholinos specifically reduce translation of Hipk1. (A) Target sequences of Hipk1MO1 and Hipk1MO2 in the 5′UTR of X. laevis hipk1. (B, C) In vitro reticulocyte lysate transcription and translation reactions performed in the presence of 35S with plasmids encoding X. laevis Hipk1 and Dsh proteins; products separated by SDS-PAGE. Both Hipk1MO1 and Hipk1MO2 reduce Hipk1 protein in a dose-dependent manner. Based on its superior specificity as judged by less off-target effects on levels of the Dsh protein, Hipk1MO1 (B) was primarily used for Hipk1 loss-of-function experiments. Hipk1MO2 (C) was used at lower doses to corroborate findings obtained with Hipk1MO1.
(1.21 MB TIF)

Figure S5 Hipk1MO2 inhibits Keller explant elongation. 20 ng per embryo of the control morpholino (CoMO) or Hipk1MO2 were injected into the DMZ. Keller explant sandwiches were prepared and cultured under glass until Stage 19 as described for Figure 6B.
(5.13 MB TIF)

Table S1 Supplemental Table 1 lists results for Hipk1 mRNA injections into the DMZ of X. laevis embryos.
(0.02 MB PDF)

Table S2 Supplemental Table 2 lists results for Hipk1 morpholino injections into the DMZ of X. laevis embryos
(0.02 MB PDF)
