# Supplementary material for: Modulation of the β-Catenin Signaling Pathway by the Dishevelled-Associated Protein Hipk1
Source: PLoS One. 2009 Feb 2;4(2):e4310. doi: 10.1371/journal.pone.0004310 (PMC2629544; doi:10.1371/journal.pone.0004310)
Supplement: Figure S1 — Multiple sequence alignment of Hipk-family members and X. laevis hipk1. Residues identical to the X. laevis protein are shaded gray, and introduced gaps represented by a dash (-). Conserved domains defined in the literature are boxed and labeled. Sequence corresponding to the partial-length clone recovered in the yeast 2-hybrid screen is labeled as the “Dishevelled Interacting Region” (DIR). The conserved lysine in the ATP-binding portion of the kinase domain, which was mutated to create (K/R)Hipk1, is indicated in red. Accession numbers are listed in the Materials and Methods. (0.08 MB PDF) [file pone.0004310.s001.pdf]

Figure S1

```
                  1                                                50
HIPK1_Hs     (1) -------MASQLQVFSP--PSVSSSAFCSAKKLKIEPS-GWDVSGQSSND
HIPK1_Mm     (1) -------MASQLQVFSP--PSVSSSAFCSAKKLKIEPS-GWDVSGQSSND
HIPK1_Gg     (1) -------MASQLQVFSP--PSVSSSAFCSAKKLKVEPS-VWDVSGQSSSD
HIPK1_Xt     (1) -------MASQLQVFSP--SSVSSSAFCSAKKLKVEPS-GWDVTGQGSSN
HIPK1_Xl     (1) -------MASQLQVFSP--SSVSSSAFYSAKKLKVEPS-GWDVTGQGSSS
HIPK2_Hs     (1) MAPVYEGMASHVQVFSP--HTLQSSAFCSVKKLKIEPSSNWDMTGYGSHS
HIPK2_Mm     (1) MAPVYEGMASHVQVFSP--HTLQSSAFCSVKKLKVEPSSNWDMTGYGSHS
HIPK2_Gg     (1) -------MASHVQVFSP--HTLQSSAFCSVKKLKVEPSSNWDMTGYGTHS
HIPK2_Xt     (1) -------MASHLQIFSS--HGLPLRTFCKVKKPKLELSPGWDMTGYGCHS
HIPK3_Hs     (1) -------MASQVLVYPPYVYQTQSSAFCSVKKLKVEPS--SCVFQERNYP
HIPK3_Mm     (1) -------MASQVLVYPPYVYQTQSSAFCSVKKLKVEPS--GCVFQERTYP
HIPK3_Gg     (1) -------MASQVLVYPPYVYQTQSSAFCSVKKLKLEPS--SCVYHERAYP
HIPK3_Xt     (1) -------MASQVLVYPPYVYQTQSSAFCSIKKLKVEPS--SCLYNKRSYQ

                  51                                              100
HIPK1_Hs    (41) KYYTHSKTLPATQGQANSSHQ---VANFNIPAYDQGLLLPAPAVEHIVVT
HIPK1_Mm    (41) KYYTHSKTLPATQGQASSSHQ---VANFNLPAYDQGLLLPAPAVEHIVVT
HIPK1_Gg    (41) KYYTHSKNLPAAQGQASSSHQ---VANFSVPAYDQNLLLPAPSVEHIVVT
HIPK1_Xt    (41) KYYTHTKNLPVAQGHASSSHQ---VAGFSLPAYEPNLLIPAAAAEHIVVT
HIPK1_Xl    (41) KYYTR--NLPVAQGHASSSRQ---VVGFSLPAYEPNLLIPAAAAEHIVVT
HIPK2_Hs    (49) KVYSQSKNIPLS-QPATTTVSTSLPVPNPSLPYEQTIVFPGSTGHIVVTS
HIPK2_Mm    (49) KVYSQSKNIPPS-QPASTTVSTSLPIPNPSLPYEQTIIFPGSTGHIVVTS
HIPK2_Gg    (42) KVYSQSKSVQSSQAAAAAVNASLQIPNPSIPYEQTIIFPASTGHIVVTS
HIPK2_Xt    (42) KVYNSGKNSSTS------GQP-VLSHASLLQSYEQTVIFPTSAGHIVVTS
HIPK3_Hs    (42) RTYVNGRNFGNSHPPTKG-SAFQTKIPFNRPRGHNFSLQTSAVVLKNTAG
HIPK3_Mm    (42) QIHVNGRNFGNSHPSTKG-SAFQTNIPFTKPRGHSFSLQAGAIVVKDTAG
HIPK3_Gg    (42) QIYVNGKNFG-ISPHRVS-TFLQTKNPFDRPRGQNVLLQSNAVALKNIAG
HIPK3_Xt    (42) RTDLNARSIGFAQPSNKNRAPFQTKNTSDKPRHQAAVSKTSPVTLNTNTG

                  101                                             150
HIPK1_Hs    (88) AADSSG--SAATSTFQSS-QTLTHRSNVSLLEPYQKCGLKRKSEEVDS-N
HIPK1_Mm    (88) AADSSG--SAATATFQSS-QTLTHRSNVSLLEPYQKCGLKRKSEEVES-N
HIPK1_Gg    (88) AADSTG--SSATASFQNS-QTLTHRTNLSLLEPYQKCGLKRKSEEVDS-N
HIPK1_Xt    (88) AADSTD--SGPTTSFQNS-QILTHRSNVPLLDPYQKCGLKRKSEEVDS-N
HIPK1_Xl    (86) AADSTD--SGPTTSFQNS-QILTHRSNVPLLDPYQKCGLKRKSEEVDS-N
HIPK2_Hs    (98) ASSTS----VTGQVLGGP-HNLMRRSTVSLLDTYQKCGLKRKSEEIEN-T
HIPK2_Mm    (98) ASSTS----VTGQVLGGP-HNLMRRSTVSLLDTYQKCGLKRKSEEIEN-T
HIPK2_Gg    (92) ANSTSGVVAVSGQTLGGP-HNLMRRSTVSLLDTYQKCGLKRKSEEIEN-T
HIPK2_Xt    (85) SSNGSS--AATGQALGGPQTNLMRRSTVSLLDTYQKCGLKRKCEELEN-N
HIPK3_Hs    (91) ATKVIAAQAQQAHVQAPQ--IGAWRNRLHFLEGPQRCGLKRKSEELDNHS
HIPK3_Mm    (91) ATKVLAAQAQQAGVEAPR--AVVWRNRLHFLEGPQRCGLKRKSEELENHS
HIPK3_Gg    (90) ATKALAAQAQQAQLEAPR--SGTQGSRADILEGPQRCGLKRKSEELDNQS
HIPK3_Xt    (92) ASGDLAAQAQQDPKEELT--VGTPESGSDLLDAHQRCGLKRKSKDFENQN

                  151                                             200
HIPK1_Hs   (134) GSVQIIEEHPPLMLQNRTVVGAAATTTTVTTK--SSSSSGEGDYQLVQHE
HIPK1_Mm   (134) GSVQIIEEHPPLMLQNRTVVGAAATTTTVTTK--SSSSSGEGDYQLVQHE
HIPK1_Gg   (134) GSVQIIEEHPPLMLQNRPAVGAAATTTTVTTK--SSNSSGEGDYQLVQHE
HIPK1_Xt   (134) GSVQIIDERPPLMLQNRTVVGAAATTTTVTTK--SSSSNGEGDYQLVQHE
HIPK1_Xl   (132) GSVQIIDERPPLMLQNRTVVGAAATTTTVTTK--SSSSSGGGDYQLVQHE
HIPK2_Hs   (142) SSVQIIEEHPPMIQNNASGATVATATTSTATSK-NSGSNSEGDYQLVQHE
HIPK2_Mm   (142) SSVQIIEEHPPMIQNNASGATVATATTSTATSK-NSGSNSEGDYQLVQHE
HIPK2_Gg   (140) SSVQIIEEHPPMIQNNASGATVATATTSTATSK-NSGSNSEGDYQLVQHE
HIPK2_Xt   (132) SSVQIVDEHPPVTQNNASGATAATTSTATSK---NSGSNSEGDYQLVQHE
HIPK3_Hs   (139) SAMQIVDELSILPAMLQTNMGNPVTVVTATTGSKQNCTTGEGDYQLVQHE
HIPK3_Mm   (139) GAMQIVDELSILPAMLQTNMGNPVTVVTATTGSKQNCTSGEGDYQLVQHE
HIPK3_Gg   (138) STMQIVDELSILPAMLQTNVGNPVTVVTTAATSKQTGTSGDGDYQLVQHE
HIPK3_Xt   (140) GTMQIVDELSILPAMLPNNAANQVTVVATTGTSKQSTASGDGDYQLMLHE
```

ATP-binding/ Kinase Domain →

```
                  201                                              250
HIPK1_Hs   (182) ILCSMTNSYEVLEFLGRGTFGQVAKCWKRSTKEIVAIKILKNHPSYARQG
HIPK1_Mm   (182) ILCSMTNSYEVLEFLGRGTFGQVAKCWKRSTKEIVAIKILKNHPSYARQG
HIPK1_Gg   (182) ILCSMTNSYEVLEFLGRGTFGQVAKCWKRSTKEIVAIKILKNHPSYARQG
HIPK1_Xt   (182) ILCSMTNSYEVLEFLGRGTFGQVAKCWKRSTKEIVAIKILKNHPSYARQG
HIPK1_Xl   (180) ILCSMTNSYEVLEFLGRGTFGQVAKCWKRSTKEIVAIKILKNHPSYARQG
HIPK2_Hs   (191) VLCSMTNTYEVLEFLGRGTFGQVVKCWKRGTNEIVAIKILKNHPSYARQG
HIPK2_Mm   (191) VLCSMTNTYEVLEFLGRGTFGQVVKCWKRGTNEIVAIKILKNHPSYARQG
HIPK2_Gg   (189) VLCSMTNTYEVLEFLGRGTFGQVVKCWKRGTNEIVAIKILKNHPSYARQG
HIPK2_Xt   (179) VLCSMTNTYEVLEFLGRGTFGQVVKCWKRGTNEIVAIKILKNHPSYARQG
HIPK3_Hs   (189) VLCSMKNTYEVLDFLGRGTFGQVVKCWKRGTNEIVAIKILKNHPSYARQG
HIPK3_Mm   (189) VLCSMKNTYEVLDFLGRGTFGQVVKCWKRGTNEIVAIKILKNHPSYARQG
HIPK3_Gg   (188) VLCSVKNTYEVLDFLGRGTFGQVVKCWKRGTNEIVAIKILKNHPSYARQG
HIPK3_Xt   (190) VLCSVKNTYEVLDFLGRGTFGQVVKCWRRGTNEVVAVKILKNHPSYARQG

                  251                                              300
HIPK1_Hs   (232) QIEVSILSRLSSENADEYNFVRSYECFQHKNHTCLVFEMLEQNLYDFLKQ
HIPK1_Mm   (232) QIEVSILSRLSSENADEYNFVRSYECFQHKNHTCLVFEMLEQNLYDFLKQ
HIPK1_Gg   (232) QIEVSILSRLSSENADEYNFVRSYECFQHKNHTCLVFEMLEQNLYDFLKQ
HIPK1_Xt   (232) QIEVSILSRLSSENADEYNFVRSYECFQHKNHTCLVFEMLEQNLYDFLKQ
HIPK1_Xl   (230) QIEVSILSRLSSENADEYNFVRSYECFQHKNHTCLVFEMLGQNLYDFLKQ
HIPK2_Hs   (241) QIEVSILARLSTESADDYNFVRAYECFQHKNHTCLVFEMLEQNLYDFLKQ
HIPK2_Mm   (241) QIEVSILARLSTESADDYNFVRAYECFQHKNHTCLVFEMLEQNLYDFLKQ
HIPK2_Gg   (239) QIEVSILARLSTESADDYNFVRAYECFQHKNHTCLVFEMLEQNLYDFLKQ
HIPK2_Xt   (229) QIEVSILARLSTESADDYNFVRAYECFQHKNHTCLVFEMLEQNLYDFLKQ
HIPK3_Hs   (239) QIEVSILARLSTENADEYNFVRAYECFQHRNHTCLVFEMLEQNLYDFLKQ
HIPK3_Mm   (239) QIEVSILARLSTENADEYNFVRAYECFQHRNHTCLVFEMLEQNLYDFLKQ
HIPK3_Gg   (238) QIEVSILARLSTENADEFNFVRAYECFQHRNHTCLVFEMLEQNLYDFLKQ
HIPK3_Xt   (240) QIEMGILARLSNENADEFNFVRAYECFQHRNHTCLVFEMLEQNLYDFLKQ

                  301                                              350
HIPK1_Hs   (282) NKFSPLPLKYIRPILQQVATALMKLKSLGLIHADLKPENIMLVDPVRQPY
HIPK1_Mm   (282) NKFSPLPLKYIRPILQQVATALMKLKSLGLIHADLKPENIMLVDPVRQPY
HIPK1_Gg   (282) NKFSPLPLKYIRPILQQVATALMKLKSLGLIHADLKPENIMLVDPARQPY
HIPK1_Xt   (282) NKFSPLPLKYIRPILQQVATALMKLKSLGLIHADLKPENIMLVDPVRQPY
HIPK1_Xl   (280) NKFSPLPLKYIRPILQQVATALMKLKSLGLIHADLKPENIMLLDPVRQPY
HIPK2_Hs   (291) NKFSPLPLKYIRPVLQQVATALMKLKSLGLIHADLKPENIMLVDPSRQPY
HIPK2_Mm   (291) NKFSPLPLKYIRPVLQQVATALMKLKSLGLIHADLKPENIMLVDPSRQPY
HIPK2_Gg   (289) NKFSPLPLKYIRPILQQVATALMKLKSLGLIHADLKPENIMLVDPSRQPY
HIPK2_Xt   (279) NKFSPLPLKYIRPVLQQVGTALMKLKSLGLIHADLKPENIMLVDPSRQPY
HIPK3_Hs   (289) NKFSPLPLKVIRPILQQVATALKKLKSLGLIHADLKPENIMLVDPVRQPY
HIPK3_Mm   (289) NKFSPLPLKVIRPVLQQVATALKKLKSLGLIHADLKPENIMLVDPVRQPY
HIPK3_Gg   (288) NKFSPLQLKVIRPILQQVATALKKLKSLGLIHADLKPENIMLVDPVRQPY
HIPK3_Xt   (290) NKFSPLPLKVIRAILQQVATALKKLKSLGLIHADLKPENIMLVDPVRQPY

                  351                                              400
HIPK1_Hs   (332) RVKVIDFGSASHVSKAVCSTYLQSRYYRAPEIILGLPFCEAIDMWSLGCV
HIPK1_Mm   (332) RVKVIDFGSASHVSKAVCSTYLQSRYYRAPEIILGLPFCEAIDMWSLGCV
HIPK1_Gg   (332) RVKVIDFGSASHVSKAVCSTYLQSRYYRAPEIILGLPFCEAIDMWSLGCV
HIPK1_Xt   (332) RVKVIDFGSASHVSKAVCSTYLQSRYYRAPEIILGLPFCEAIDMWSLGCV
HIPK1_Xl   (330) RMKVIDFGSASHVSKAVCSTYLQSRYYRAPEIILGLPFCEAIDMWSLGCV
HIPK2_Hs   (341) RVKVIDFGSASHVSKAVCSTYLQSRYYRAPEIILGLPFCEAIDMWSLGCV
HIPK2_Mm   (341) RVKVIDFGSASHVSKAVCSTYLQSRYYRAPEIILGLPFCEAIDMWSLGCV
HIPK2_Gg   (339) RVKVIDFGSASHVSKAVCSTYLQSRYYRAPEIILGLPFCEAIDMWSLGCV
HIPK2_Xt   (329) RVKVIDFGSASHVSKAVCSTYLQSRYYRAPEIILGLPFCEAIDMWSLGCV
HIPK3_Hs   (339) RVKVIDFGSASHVSKTVCSTYLQSRYYRAPEIILGLPFCEAIDMWSLGCV
HIPK3_Mm   (339) RVKVIDFGSASHVSKTVCSTYLQSRYYRAPEIILGLPFCEAIDMWSLGCV
HIPK3_Gg   (338) RVKVIDFGSASHVSKTICSTYLQSRYYRAPEIILGLPFCEAIDMWSLGCV
HIPK3_Xt   (340) RVKVIDFGSASYVSKTVCSTYLQSRYYRAPEVILGLPFCEAIDMWSLGCV
```

```
                  401                                               450
HIPK1_Hs   (382) IAELFLGWPLYPGASEYDQIRYISQTQGLPAEYLLSAGTKTTRFFNRDPN
HIPK1_Mm   (382) IAELFLGWPLYPGASEYDQIRYISQTQGLPAEYLLSAGTKTTRFFNRDPN
HIPK1_Gg   (382) IAELFLGWPLYPGASEYDQIRYISQTQGLPAEYLLSAGTKTSRFFNRDPN
HIPK1_Xt   (382) IAELFLGWPLYPGASEYDQIRYISQTQGLPAEYLLSAGTKTSRFFNRDPD
HIPK1_Xl   (380) IAELFLGWPLYPGASEYDQIRYISQTQGLPAEYLLSAGTKTSRFFNRDPD
HIPK2_Hs   (391) IAELFLGWPLYPGASEYDQIRYISQTQGLPAEYLLSAGTKTTRFFNRDTD
HIPK2_Mm   (391) IAELFLGWPLYPGASEYDQIRYISQTQGLPAEYLLSAGTKTTRFFNRDTD
HIPK2_Gg   (389) IAELFLGWPLYPGASEYDQIRYISQTQGLPAEYLLSAGTKTTRFFNRDTD
HIPK2_Xt   (379) IAELFLGWPLYPGASEYDQIRYISQTQGLPAEYLLSSGTKTTRFFNRDGD
HIPK3_Hs   (389) IAELFLGWPLYPGALEYDQIRYISQTQGLPGEQLLNVGTKSTRFFCKETD
HIPK3_Mm   (389) IAELFLGWPLYPGALEHDQIRYISQTQGLPGEQLLNVGTKSTRFFCRETD
HIPK3_Gg   (388) IAELFLGWPLYPGALEYDQIRYISQTQGLPGEQLLSMGTKTARFFCRETD
HIPK3_Xt   (390) IAELFLGWPLYPGAQEYDQIRYISQTQGLPGDSLINAGTKTSRFFCREPD

                  451                                               500
HIPK1_Hs   (432) LGYPLWRLKTPEEHELETGIKSKEARKYIFNCLDDMAQVNMSTDLEGTDM
HIPK1_Mm   (432) LGYPLWRLKTPEEHELETGIKSKEARKYIFNCLDDMAQVNMSTDLEGTDM
HIPK1_Gg   (432) LGYPLWRLKTPEEHELETGIKSKEARKYIFNCLDDMAQVNMSTDLEGTDM
HIPK1_Xt   (432) LGYPLWRLKAPDEHEMETGIKSKEARKYIFNCLDDMAQVNMSTDLEGTDM
HIPK1_Xl   (430) LGYPLWRLKAPDEHEVETGIKSKEARKYIFNCLDDMAQVNMSTDLEGTDM
HIPK2_Hs   (441) SPYPLWRLKTPDDHEAETGIKSKEARKYIFNCLDDMAQVNMTTDLEGSDM
HIPK2_Mm   (441) SPYPLWRLKTPDDHEAETGIKSKEARKYIFNCLDDMAQVNMTTDLEGSDM
HIPK2_Gg   (439) SPYPLWRLKTPDDHEAETGIKSKEARKYIFNCLDDMAQVNMTTDLEGSDM
HIPK2_Xt   (429) SPYPLWRLKTPEDHENETGIKSKEARKYIFNCLDDMAQVNMASDLEGSDM
HIPK3_Hs   (439) MSHSGWRLKTLEEHEAETGMKSKEARKYIFNSLDDVAHVNTVMDLEGSDL
HIPK3_Mm   (439) MSHSGWRLKTLEEHEAETGMKSKEARKYIFNSLDDIVHVNTVMDLEGGDL
HIPK3_Gg   (438) APYSSWRLKTLEEHEAETGMKSKEARKYIFNSLDDIVHVNMVMDLEGSDL
HIPK3_Xt   (440) APYPSWRLKTLEEHEAETGIKSKETRKYIFNNLDEIVHVNMMVDLDGSDL

                  501                    ←——Kinase Domain           550
HIPK1_Hs   (482) LAEKADRREYIDLLKKMLTIDADKRITPLKTLNHQFVTMTHLLDFPHSNH
HIPK1_Mm   (482) LAEKADRREYIDLLKKMLTIDADKRITPLKTLNHQFVTMSHLLDFPHSSH
HIPK1_Gg   (482) LAEKADRREYIDLLKKMLTIDADKRITPLKTLNHPFVTMTHLLDFPHSNH
HIPK1_Xt   (482) LAEKADRREYIDLLKKMLTIDADKRITPLKTLNHPFVTMTHLLDFPHSNH
HIPK1_Xl   (480) LAEKADRREYIDLLKKMLTIDADKRITPLKTLNHPFVTMTHLLDFPHSNH
HIPK2_Hs   (491) LVEKADRREFIDLLKKMLTIDADKRITPIETLNHPFVTMTHLLDFPHSTH
HIPK2_Mm   (491) LVEKADRREFIDLLKKMLTIDADKRVTPIETLNHPFVTMTHLLDFPHSAH
HIPK2_Gg   (489) LVEKADRREFIDLLKKMLTIDADKRITPIETLNHPFVTMTHLLDFPHSTH
HIPK2_Xt   (479) LVEKADRREFIDLLKKMLTIDADKRITPIETLNHPFVTMTHLLDFPHSSH
HIPK3_Hs   (489) LAEKADRREFVSLLKKMLLIDADLRITPAETLNHPFVNMKHLLDFPHSNH
HIPK3_Mm   (489) LAEKADRREFVNLLKKMLLIDADLRITPIETLNHPFVNMKHLLDFPHSNH
HIPK3_Gg   (488) LAEKADRREFVSLLKKMLLIDADLRITPAETLNHSFVTMKHLLDFPHSNQ
HIPK3_Xt   (490) MAEKADRREFVALLKKMLMIDADKRISPADTLSQSFITMKHLLDFPHSNH

                  551             Homeodomain-Interacting Domain →   600
HIPK1_Hs   (532) VKSCFQNMEICKRRVHMYDTVSQIKSPFTTHVAPNTSTNLTMSFSNQLNT
HIPK1_Mm   (532) VKSCFQNMEICKRRVHMYDTVSQIKSPFTTHVAPNTSTNLTMSFSNQLNT
HIPK1_Gg   (532) VKSCFQNMEICKRRVNMYDTVNQIKSPFTTHVAPNTSTNLTMSFNNQLNT
HIPK1_Xt   (532) VKSCFQNMEICKRRSNMYDTVNQIKSPFTTHVAPNTSTNLTMSFNNQLNS
HIPK1_Xl   (530) VKSCFQNMEICKRRSNMYDTVNQIKSPFTTHVAPNTSTNLTMSFNNQLNS
HIPK2_Hs   (541) VKSCFQNMEICKRRVNMYDTVNQSKTPFITHVAPSTSTNLTMTFNNQLTT
HIPK2_Mm   (541) VKSCFQNMEICKRRVNMYDTVNQSKTPFITHVAPSTSTNLTMTFNNQLTT
HIPK2_Gg   (539) VKSCFQNMEICKRRVNMYDTVNQSKTPFITHVAPSTSTNLTMTFNNQLNT
HIPK2_Xt   (529) VKSCFQNMEICKRRVNMYDTVNHSKTPFITHVAPSTSTNLTMTFSNQLNT
HIPK3_Hs   (539) VKSCFHIMDICKSHLNSCDTNNHNKTSLLRPVASSSTATLTANFT-KIGT
HIPK3_Mm   (539) VKSCFHIMDICKSPS-SCETNNHSKMSLLRPVASNGTAALAANFT-KVGT
HIPK3_Gg   (538) VKSCFHIMDVCKCRSNLYDLSNRNKTSLMRPVASGSAANLTASFT-KIGP
HIPK3_Xt   (540) VKSCFYIMDVCKARPNVGDTANHNKTSIVRPITSTNIGSMSAGFT-KIGT
```

```
                601                                              650
HIPK1_Hs   (582) VHNQASVLASSSTAAAATLSLANSDVSLLNYQSALYPSSAAPVPGVAQQG
HIPK1_Mm   (582) VHNQASVLASSSTAAAATLSLANSDVSLLNYQSALYPSSAAPVPGVAQQG
HIPK1_Gg   (582) VHNQASVLASN-STAAATLSLANSDVSLLNYQSALYPSSAAPVAGVAQQS
HIPK1_Xt   (582) VHNQASVLASS-STAAATLSLANSDVSLLNYQSALYPPAAGPVTGVTQQS
HIPK1_Xl   (580) VHNQASVLASS-STAAATLSLANSDVSLLNYQSALYPPAAGPVTGVTQQS
HIPK2_Hs   (591) VHN---------------------------------QPSAASMAAVAQRS
HIPK2_Mm   (591) VHNQAP----T--TSSATLSLANPEVSILNYQSALYQPSAASMAAVAPRS
HIPK2_Gg   (589) VHNQTTNLAPT--SSSATISLANPEVSILNYQSALYQPSAASMAAVAQRS
HIPK2_Xt   (579) VHS---------------------------------QPTAASMAAVAQRT
HIPK3_Hs   (588) LRSQ---------------------------------ALTTSAHSVVHHG
HIPK3_Mm   (587) LRSQ---------------------------------ALTTSAHSVVHHG
HIPK3_Gg   (587) LRSQ---------------------------------ALTASAHSVLHQG
HIPK3_Xt   (589) IRGQ---------------------------------ALTTSGHSVIHHG

                651                                              700
HIPK1_Hs   (632) VSLQPGTTQICTQTDPFQQTFIVCPPAFQTG-LQATTKHSGFPVRMDNAV
HIPK1_Mm   (632) VSLQPGTTQICTQTDPFQQTFIVCPPAFQTG-LQATTKHSGFPVRMDNAV
HIPK1_Gg   (631) VSLQPGTTQICTQTDPFQQTFIVCPPAFQTG-LQATTKHSGFPVRMENAV
HIPK1_Xt   (631) VSLQPGTTQLCTQTDPFQQTFIVCPPAFQAAGLQATTKHSGFPVRMDNSV
HIPK1_Xl   (629) VSLQPGTTQLCTQTDPFQQTFIVCPPAFQAAGLQATTKHSGFPVRMDNSV
HIPK2_Hs   (608) MPLQTGTAQICARPDPFQQALIVCPPGFQGL-QASPSKHAGYSVRMENAV
HIPK2_Mm   (635) MPLQTGTAQICARPDPFQQALIVCPPGFQGL-QASPSKHAGYSVRMENAV
HIPK2_Gg   (637) MPLQTGTAQICARPDPFQQALIVCPPGFQGL-QASPSKHAGYSVRMENAV
HIPK2_Xt   (596) MPLQTGTAQLCARPDPFQQALIVCPPAFQGL-QTSPSKHGGYSVRMENTV
HIPK3_Hs   (605) IPLQAGTAQFGCG-DAFQQTLIICPPAIQGI-PATHGKPTSYSIRVDNTV
HIPK3_Mm   (604) IPLQAGTAQFGCG-DAFHQTLIICPPAIQGI-PAAHGKPTSYSIRVDNTV
HIPK3_Gg   (604) IPLQAGTAQFGCS-DIFQQTLIICPPTLQGI-TTNHSKPTSFSLRVDNAV
HIPK3_Xt   (606) IPLQAGSAPFGCN-DAFQQTLILCPPAIQGM-PPNHGKPTSYSVRVDNAL

                701                                              750
HIPK1_Hs   (681) PIVPQAPAAQPLQIQSGVLTQGSCTPLMVATLHPQVATITPQYAVPFTLS
HIPK1_Mm   (681) PIVPQAPAAQPLQIQSGVLTQGSCTPLMVATLHPQVATITPQYAVPFTLS
HIPK1_Gg   (680) PIVPQAPAAQPLQIQSGVLTQGSCTPLMVATLHPQVATITPQYAVPFTLN
HIPK1_Xt   (681) PIVPQAPAAQPLQIQSGVLTQ-----------------------------
HIPK1_Xl   (679) PIVPQAPAAQPPQIQSGVLTQ-----------------------------
HIPK2_Hs   (657) PIVTQAPGAQPLQIQPGLLAQ-----------------------------
HIPK2_Mm   (684) PIVTQAPGAQPLQIQPGLLAQ-----------------------------
HIPK2_Gg   (686) PIVTQAPGAQPLQIQPGLLAQ-----------------------------
HIPK2_Xt   (645) PLVTQTAGAQALQIQPGLLTQ-----------------------------
HIPK3_Hs   (653) PLVTQAPAVQPLQIRPGVLS------------------------------
HIPK3_Mm   (652) PLVTQAPAVQPLQIRPGVLSQ-----------------------------
HIPK3_Gg   (652) PLVTQAHAIQPLQIRAGILS------------------------------
HIPK3_Xt   (654) PLVTQAPAIQPLQIRAGVLT------------------------------

                751                                              800
HIPK1_Hs   (731) CAAGRPALVEQTAAVLQAWPGGTQQILLPSTWQQLPGVALHNSVQPTAMI
HIPK1_Mm   (731) CAAGRPALVEQTAAVLQAWPGGTQQILLPSAWQQLPGVALHNSVQPAAVI
HIPK1_Gg   (730) CAAGRPALVEQTAAVLQAWPGGTQQILLPSTWQQLPGVALHNSVQPAAVI
HIPK1_Xt   (702) -----------------AWPGGTQQILLPSTWQQLPGVALHNSVQPTAMI
HIPK1_Xl   (700) -----------------AWPGGTQQILLPSTWQQLPGVALHNSVQPSAMI
HIPK2_Hs   (678) ----------------QAWPSGTQQILLPPAWQQLTGVATHTSVQHATVI
HIPK2_Mm   (705) ----------------QAWPGGAQQILLPPAWQQLTGVATHTSVQHAAVI
HIPK2_Gg   (707) ----------------QAWPSGTQQILLPPAWQQLTGVATHTSVQHATVI
HIPK2_Xt   (666) ----------------QAWPSGTQQILLPPAWQQLAGVAAHTSVQHATVI
HIPK3_Hs   (673) ----------------QTWSGRTQQMLVP-AWQQVTPLAPATTTLTSESV
HIPK3_Mm   (673) ----------------QTWSGRTQQMLIP-AWQQVTPMAPAAATLTSEGM
HIPK3_Gg   (672) ----------------QTWSNHTQQILVP-AWQQVAPVAAPATSLASDPV
HIPK3_Xt   (674) ----------------QAWSNGTQPILVP-AWQQMTTIAPTASSLASDTM
```

```
                    ← Homeodomain-Interacting Domain
                 801                                                850
HIPK1_Hs    (781) PEAMGSGQQLADWR-NAHSHGNQYSTIMQQPSLLTNHVTLATAQPLNVGV
HIPK1_Mm    (781) PEAMGSSQQLADWR-NAHSHGNQYSTIMQQPSLLTNHVTLATAQPLNVGV
HIPK1_Gg    (780) PETIGSSQQLADWR-NAHSHGNQYSTLMQQPSLLTNHVTLATAQPLNVGV
HIPK1_Xt    (735) PETIGNNQQLTDWR-NAHSHGNQYSTLMQQPSLLANHVTLAAAQPLNVGV
HIPK1_Xl    (733) PETIGNNQQLTDWSRNAHSHGNQYSTLMQQPSLLANHVTLAAAQPLNVGV
HIPK2_Hs    (712) PETMAGTQQLADWR-NTHAHGSHYNPIMQQPALLTGHVTLPAAQPLNVGV
HIPK2_Mm    (739) PETMAGTQQLADWR-NTHAHGSHYNPIMQQPALLTGHVTLPAAQPLNVGV
HIPK2_Gg    (741) PESMAGTQPLADWR-NTHAHGSHYNPIMQQPALLASHVTLPAAQPVNVGV
HIPK2_Xt    (700) PDGMAGTQQLADWR-NTHAHGTHYNPIMQQPALLTGHVTLPSAQPLNVGV
HIPK3_Hs    (706) AGSH----RLGDWG-KMISCSNHYNSVMPQP-LLTNQITLSAPQPVSVGI
HIPK3_Mm    (706) AGSQ----RLGDWG-KMIPHSNHYNSVMPPP-LLTNQITLSAPQPISVGI
HIPK3_Gg    (705) AGPQ----RLGDWG-KMITQGTHYNSVMPQP-LLTHQITFSAPQPISVGI
HIPK3_Xt    (707) AGPQ----RLGDWG-KVIPHGNHYNSMIIQP-IITNQMTLSAPQPISLGI

                  851 Dsh-Interacting Region →                      900
HIPK1_Hs    (830) AHVVRQQQSSSLPSKKNKQS----------------------APV-SSK-
HIPK1_Mm    (830) AHVVRQQQSSSLPSKKNKQS----------------------APV-SSK-
HIPK1_Gg    (829) AHVVRQQQSSNVPAKKNKQP----------------------APSTAN--
HIPK1_Xt    (784) AHVVRQQQNSNTSTKKNKQQ----------------------ASQSSSK-
HIPK1_Xl    (783) AHVVRQQQNSNTSTKKNKQQ----------------------ASQKSSK-
HIPK2_Hs    (761) AHVMRQQPTSTTSSRKSKQH----------------------QSSVRNVS
HIPK2_Mm    (788) AHVMRQQPTSTTSSRKSKQH----------------------QSSVRNVS
HIPK2_Gg    (790) AHVMRQPPAAATSTRKSKQH----------------------QSAPRNAS
HIPK2_Xt    (749) AHVMRQQPTSSSSSR-KKH-----------------------HSLARNIS
HIPK3_Hs    (750) AHVVWPQPATTKKNKQCQNRGILVKLMEWEPGREEINAFSWSNSLQNTNI
HIPK3_Mm    (750) AHVVWPQPATTKKNKLCQNR--------------------S-NSLQNTNI
HIPK3_Gg    (749) AHVVWPQPAATKRNKLCQNR--------------------S-NALQNTNI
HIPK3_Xt    (751) AHVVWPQPAANKRNKLCQNR--------------------I-NAIQDIVV

                                                       SRS/PEST →
                  901                                              950
HIPK1_Hs    (856) ------SSLDVLPSQVYSLVGSSPLRTTSS----------------YNSL
HIPK1_Mm    (856) ------SSLEVLPSQVYSLVGSSPLRTTSS----------------YNSL
HIPK1_Gg    (855) ------STLETVPTQVYSLIGSSPLRSTSSS---------------SNVL
HIPK1_Xt    (811) ----ALSSLDVIPSQVYSLVGSSPLQASSS----------------YNPL
HIPK1_Xl    (810) ----ALSCLDIIPSQVYSLVGSSPLQASSS----------------YNPL
HIPK2_Hs    (789) TCEVSSSQAISSPQRSKRVKENTPPRCAMVHSSPACSTSVTCGWGDVASS
HIPK2_Mm    (816) TCEVTSSQAISSPQRSKRVKENTPPRCAMVHSSPACSTSVTCGWGDVASS
HIPK2_Gg    (818) TYEVSSSQSISSPQRSKRVKENTPPRCAMVHNSPACSTAVTCGWGDMATS
HIPK2_Xt    (776) AYEVTSSQSVNSPQRSKRVKENTPPRCAVPQNTTACVPSITCGWGDSAVG
HIPK3_Hs    (800) PHSAFISPKIINGKDVEEVSCIETQDNQNSEGEAR--NCCETSIRQDSDS
HIPK3_Mm    (779) PHSAFISPKIISGKEVEEVSCVDTQDNHTSEGEAR--TCREASVRQDSS-
HIPK3_Gg    (778) QNSALISPKIINLKAVKRISCIEAQDNHNSDGQES--NCCEASVRLEPDS
HIPK3_Xt    (780) HNPVVASPKIVSSENTRAEESSALPQDNHEEKEE---EQSSSNNTQGPAP

                  951                                             1000
HIPK1_Hs    (884) VPVQDQHQPIIIPDTPSPPVSVITIRSDTDEEEDN-----KYKPSSSGLK
HIPK1_Mm    (884) VPVQDQHQPIIIPDTPSPPVSVITIRSDTDEEEDN-----KYKPNSSSLK
HIPK1_Gg    (884) VPVQEQHQPIVIPDTPSPPVSVITIRSDTDEEEDS-----KYKPASLGMK
HIPK1_Xt    (841) LPLQEQRQPIVIPDTPSPPVSVITIRSDTDEEEDS-----KYKPTNSGMK
HIPK1_Xl    (840) LPLQEQRQPIVIPDTPSPPVSVITIRSDTDEEEDS-----KYKPTNSGME
HIPK2_Hs    (839) TTRERQRQTIVIPDTPSPTVSVITISSDTDEEEEQ-----KHAPTSTVSK
HIPK2_Mm    (866) TTRERQRQTIVIPDTPSPTVSVITISSDTDEEEEQ-----KHAPTSTVSK
HIPK2_Gg    (868) TTRERQRQPIIIPDTPSPAVSVITISSDTDEEEEQ-----KHAPTSTLSK
HIPK2_Xt    (826) GSHNRQRQTIVIPDTPSPAVSVITISSDTDEEEEQ-----KHVAPSLP-K
HIPK3_Hs    (848) SVSDKQRQTIIIADSPSPAVSVITISSDTDEEETSQRHSLRECKGSLDCE
HIPK3_Mm    (826) -VSDKQRQTIIIADSPSPAVSVITISSDSDDEETSPRPSLRECKGSLDCE
HIPK3_Gg    (826) SLSNKQRQAIFIAGSPSPAVSVITISSDTDEDDLGQTRSLRECKGSLDCE
HIPK3_Xt    (827) DTSIQQQQAIVIGDSPSPTVSVITINSDTDEDDPAQTLSLRECKRSLDCD
```

```
                                                    ← SRS/PEST
                  1001
HIPK1_Hs   (929) PRSNVISYVTVNDSPDSDSSLSSPYSTDTLSALRGNSGSVLEGPGRVVAD
HIPK1_Mm   (929) ARSNVISYVTVNDSPDSDSSLSSPHPTDTLSALRGNSGTLLEGPGRPAAD
HIPK1_Gg   (929) QRSNVISYVTVNDSPDSDSSLNSPYATDPLSSLR-STGGALELPSRGAAD
HIPK1_Xt   (886) LRSNVISYVTVNDSPDSDSSVSSPYLADRHTSVNGVTGVFDMSNSRTAHS
HIPK1_Xl   (885) LRSNVISYVTVNDSPDSDSSASSPCLADHHSSVNAVTGVFDISNHRAAHS
HIPK2_Hs   (884) QRKNVISCVTVHDSPYSDSSSNTSPYSVQQRAGHNNANAFDTKGSLENHC
HIPK2_Mm   (911) QRKNVISCVTVHDSPYSDSSSNTSPYSVQQRTGHNGTNTLDTKGGLENHC
HIPK2_Gg   (913) QRKNVISCVTVHDSPYSDSSSNNSPYAVQHRAGQNNGNTYDTKGVPETHC
HIPK2_Xt   (870) QRRNVISCVTVHDSPISDSSSNTSPYAASGHRRGNNTSAAESKCQTGNPC
HIPK3_Hs   (898) ACQSTLNIDRMCSLSSPDSTLSTSSSGQSSPSPCKRPNSMSDEEQESSCD
HIPK3_Mm   (875) ACQSTLNIDRMCSLSSPDSTLSTSSSGQSSPSPCKRPNSMSDDEQESGCE
HIPK3_Gg   (876) ACQNTLNIDRVCSLSSQDSTLSTSSSGQSSPSPCKRSNSMSDDEQESGCD
HIPK3_Xt   (877) TCQSALN---VCSLSSPDSTLSTSSSGQSSPSPCKRRNSISDDEPESGCD

                 1051                                          1100
HIPK1_Hs   (979) GT----------------------------GTRTIIVPPLKTQLGDCTVA
HIPK1_Mm   (979) GI----------------------------GTRTIIVPPLKTQLGDCTVA
HIPK1_Gg   (978) SS----------------------------NSRTIIVPPLKTQLNDCIVA
HIPK1_Xt   (936) QSL---------------------------NSRTIIVPPLKSQMNDCPLD
HIPK1_Xl   (935) QSS---------------------------NSRTIIVPPLKSQINDCSLN
HIPK2_Hs   (934) TG----------------------------NPRTIIVPPLKTQASEVLVE
HIPK2_Mm   (961) TG----------------------------NPRTIIVPPLKTQASEVLVE
HIPK2_Gg   (963) SG----------------------------NPRTIIVPPLKTQASEVLVE
HIPK2_Xt   (920) PA----------------------------NPRTIIVPPLKTQPSEGRLE
HIPK3_Hs   (948) TVDGSPTSDSSGHDSPFAESTFVEDTHENTELVSSADTETKPAVCSVVVP
HIPK3_Mm   (925) TVDGSPTSDSSGHDSPFAENSFVEDAHQNTELGTCAGPEAKPAVGTAVEP
HIPK3_Gg   (926) TVDGSPTSDSSGHDSPFVEDGFVANTNKNKEARASVNSETKSAVCTVVVP
HIPK3_Xt   (924) TVNGSPSSDSSGHDSPFKN-SFVRDSNQNTESRTPEKTDSKPAVCTVVVP

                1101                                          1150
HIPK1_Hs  (1001) TQASGLLS----NKTKPVASVS-GQSSGCCITPTGYRAQRGGTSAAQPLN
HIPK1_Mm  (1001) TQASGLLS----SKTKPVASVS-GQSSGCCITPTGYRAQRGGASAVQPLN
HIPK1_Gg  (1000) TQASGILSN--TSKTKPVASVS-GQSSGCCITPTGYRPHRVVANGVQPLN
HIPK1_Xt   (959) TQLTGVLSG--AAKITKSCTATNGQSSS-CITQTGCRSHR-MLNGVQPLN
HIPK1_Xl   (958) TQLTGVLSS--AAKINKTCTATNGQSSSSCITQTGQRSHR-MLNGVQPLN
HIPK2_Hs   (956) CDSLVPVN--TSHHSSSYKSKSSSNVTSTSGHSSGSSSGAITYRQQRPGP
HIPK2_Mm   (983) CDSLGPAIS-ASHHSSSFKSKSSSTVTSTSGHSSGSSSGAIAYRQQRPGP
HIPK2_Gg   (985) CDSLAPGTVTTSHHSSSYKSKSSSTVTSTSGHSSGSSSGAVAYRQQRPGA
HIPK2_Xt   (942) CERKQPDT---TSHHMYK--AKLLSSVASASHSSASSAGTAYRHSRAPGH
HIPK3_Hs   (998) PVELENGLNADEHMAN-TDSICQPLIKGRSAPGRLNQPSAVGTRQQKLTS
HIPK3_Mm   (975) PVGRESGLSVGEHMAN-TDSTCQPLRKGQPAPGKLHQPPALGARQQKPAA
HIPK3_Gg   (976) PMSLENRLRLDEQMVNTEDATCQPLKNGRSVLGRKTQSSAVGNRQQKLAS
HIPK3_Xt   (973) PIRIEN-RQFNSHRVSNKDTVCYPVIKGRSAPGRINHPNSLGSRQQKLAS

                 1151                                          1200
HIPK1_Hs  (1046) LSQNQQSSAAPTSQERSSNPAPRR----QQAFVAPLS----QAPYTFQHG
HIPK1_Mm  (1046) LSQNQQSSSASTSQERSSNPAPRR----QQAFVAPLS----QAPYAFQHG
HIPK1_Gg  (1047) LSQNQQTTVLAS-QERSGNAVPRR----QQAYVAPLTSTISQAPYTFQHS
HIPK1_Xt  (1005) LSQN-QQSAMMGTQERSMNPGSRR----QQAYVAPLP-SISQAPFTFQHS
HIPK1_Xl  (1005) LSQN-QQSALVGTQERSMNPGSRR----QQAYVAPIP-SISQAPFTFQHN
HIPK2_Hs  (1004) HFQQQQPLNLSQAQQHITTDRTGS----HRRQQAYITPTMAQAPYSFPHN
HIPK2_Mm  (1032) HFQQQQPLNLSQAQQHMAADRTGS----HRRQQAYITPTMAQAPYTFPHN
HIPK2_Gg  (1035) HFQQQQPLNLSQAQQHITTDRTGS----HRRQQAYITPTIAQAPYSFPHN
HIPK2_Xt   (987) SFQQQ-PLNLSQVSQHISTERTGS----HRRQHAYIAPTLAQAPYSFPHS
HIPK3_Hs  (1047) AFQQQH-LNFSQVQHFGSGHQEWNGNFGHRRQQAYIPTSVTSNPFTLSHG
HIPK3_Mm  (1024) AFPQQH-LNLSQVQHFGTGHQEWNGNFGHRRQQAYIPTSVTSNPFTLSHG
HIPK3_Gg  (1026) AFHQQH-LNFSQVQHFGSAPQEWNGNYAHRRQQAYIPASVASHAFSLPQG
HIPK3_Xt  (1022) AFQHQPPLNYSKVQHFGSGHQEWNGNYGHMRQQAYMQPTVASHPFTLQHG
```

```
                1201                                              1250
HIPK1_Hs  (1088) SPLHSTGHPHLAPAPAHLP----SQAHLYTYAAPTSAAALGSTSSIAHLF
HIPK1_Mm  (1088) SPLHSTGHPHLAPAPAHLP----SQPHLYTYAAPTSAAALGSTSSIAHLF
HIPK1_Gg  (1092) SPVHP--HLAAATANAHLS----SQPHMYTYAP-TTAATLGSTTSIAHLF
HIPK1_Xt  (1049) SPLHSTVHPHLAAAATATS-HLTSQPHMYTYAP-TTAASLGSTTSIAHLF
HIPK1_Xl  (1049) SPLHSTVHPHLAAAAASATSHLTSQPHMYTYAP-TTAASLGSTTSIAHLF
HIPK2_Hs  (1050) SPSHGTVHPHLAAAAAAAHLP--TQPHLYTYTA---PAALGSTGTVAHLV
HIPK2_Mm  (1078) SPSHGTVHPHLAAAA---HLP--TQPHLYTYTA---PTALGSTGTVAHLV
HIPK2_Gg  (1081) SPSHGTVHPHLAAAAAA-HLP--TQPHLYTYTT---PAALGSTGTVAHLV
HIPK2_Xt  (1032) SPSHGAVHPHLAAAH---LP---SQPHLYTYTA---PAALGSSGTVAHLV
HIPK3_Hs  (1096) SPNHTAVHAHLAGNTHLGG-----QPTLLPYPS---SATLSSAAPVAHLL
HIPK3_Mm  (1073) SPNHTAVHAHLDGSTHLGG-----QPTLLPYPS---SASLSSAAPVAHLL
HIPK3_Gg  (1075) SPNPTTVHAHLSGSTHLGG-----QPAILPYPS---SAPLSTAAPVAHLL
HIPK3_Xt  (1072) SPTHSALHAHLAGSTHIGG-----QPAILSYPP---SASLSSAAPVAHLL

                 1251 ◄ Dsh-Interacting Region                    1300
HIPK1_Hs  (1134) SPQGSSR----HAAAYTTHPSTLVHQVPVSVGPSLLTSASVAPAQYQHQF
HIPK1_Mm  (1134) SPQGSSR----HAAAYTTHPSTLVHQVPVSVGPSLLTSASVAPAQYQHQF
HIPK1_Gg  (1135) SPQGSS-----RHTQYAAHPSTLVHQVPVSVGPSLLTSANVPPAQYQHQF
HIPK1_Xt  (1097) SPQGSS-----RHTTFATHPSTLVHQVPVSVGPSLLTSANVASAQYPHQF
HIPK1_Xl  (1098) SPQGSS-----RHTTFSTHPSTLVHQVPVSVGPSLLTSANVASAQYPHQF
HIPK2_Hs  (1095) ASQGSAR----HTVQHTAYPASIVHQVPVSMGPRVLPSPTIHPSQYPAQF
HIPK2_Mm  (1120) ASQGSAR----HTVQHTAYPASIVHQVPVSMGPRVLPSPTIHPSQYPAQF
HIPK2_Gg  (1125) ASQGSAR----HAVQHTTYPASIVHQVPVSMGPRVLPSPTIHPSQYQAQF
HIPK2_Xt  (1073) ASQG-----------SARHAAYPASIVPVSMAQRLLPSPSLHPSQYQAQF
HIPK3_Hs  (1138) ASPCTSRPMLQHPTYNISHPSGIVHQVPVGLNPRLLPSPTIHQTQYKPIF
HIPK3_Mm  (1115) ASPCTSRPMLQHPTYNISHPSGIVHQVPVGINPRLLPSPTIHQTQYKPIF
HIPK3_Gg  (1117) ASPCTSRPLLQHPTYNISHPSGIVHQVPVGINPRLLPSPTIHQTQYKPIF
HIPK3_Xt  (1114) ASPCTSRPILQHPTYSLSHPSGIVHQVAVGINPRLLPSPTIHQTQFKPIF

                 1301                          1331
HIPK1_Hs  (1180) ATQSYIGSSRGSTIYTGYPLSPTKISQYSYL
HIPK1_Mm  (1180) ATQSYIGSSRGSTIYTGYPLSPTKISQYSYL
HIPK1_Gg  (1180) APQSYIGASRGSAIYTGYPLSPTKINQYSYL
HIPK1_Xt  (1142) AAQSYIGASRGSAIYTGYPLSPTNINQYSYL
HIPK1_Xl  (1143) ATPSYIGASRGSAIYTGYPLSPTNINQYSYL
HIPK2_Hs  (1141) AHQTYISASPASTVYTGYPLSPAKVNQYPYI
HIPK2_Mm  (1166) AHQTYISASPASTVYTGYPLSPAKVNQYPYI
HIPK2_Gg  (1171) AHQTYISASPASTVYTGYPLSPTKVNQYPYI
HIPK2_Xt  (1112) AHQTYIPASPTSTVYTGYPLTPPKVNQYPYI
HIPK3_Hs  (1188) PPHSYIAASP---AYTGFPLSPTKLSQYPYM
HIPK3_Mm  (1165) PPHSYIAASP---AYTGFPLSPTKVSQYPYM
HIPK3_Gg  (1167) PPHSYIAASP---AYTGFPLSPTKLSQYPFM
HIPK3_Xt  (1164) PSHSYITASP---AYAGFPMSPTKLSQYPYM
```
