# Supplementary figures and images for: Modulation of the β-Catenin Signaling Pathway by the Dishevelled-Associated Protein Hipk1
Source: PLoS One. 2009 Feb 2;4(2):e4310. doi: 10.1371/journal.pone.0004310 (PMC2629544; doi:10.1371/journal.pone.0004310)

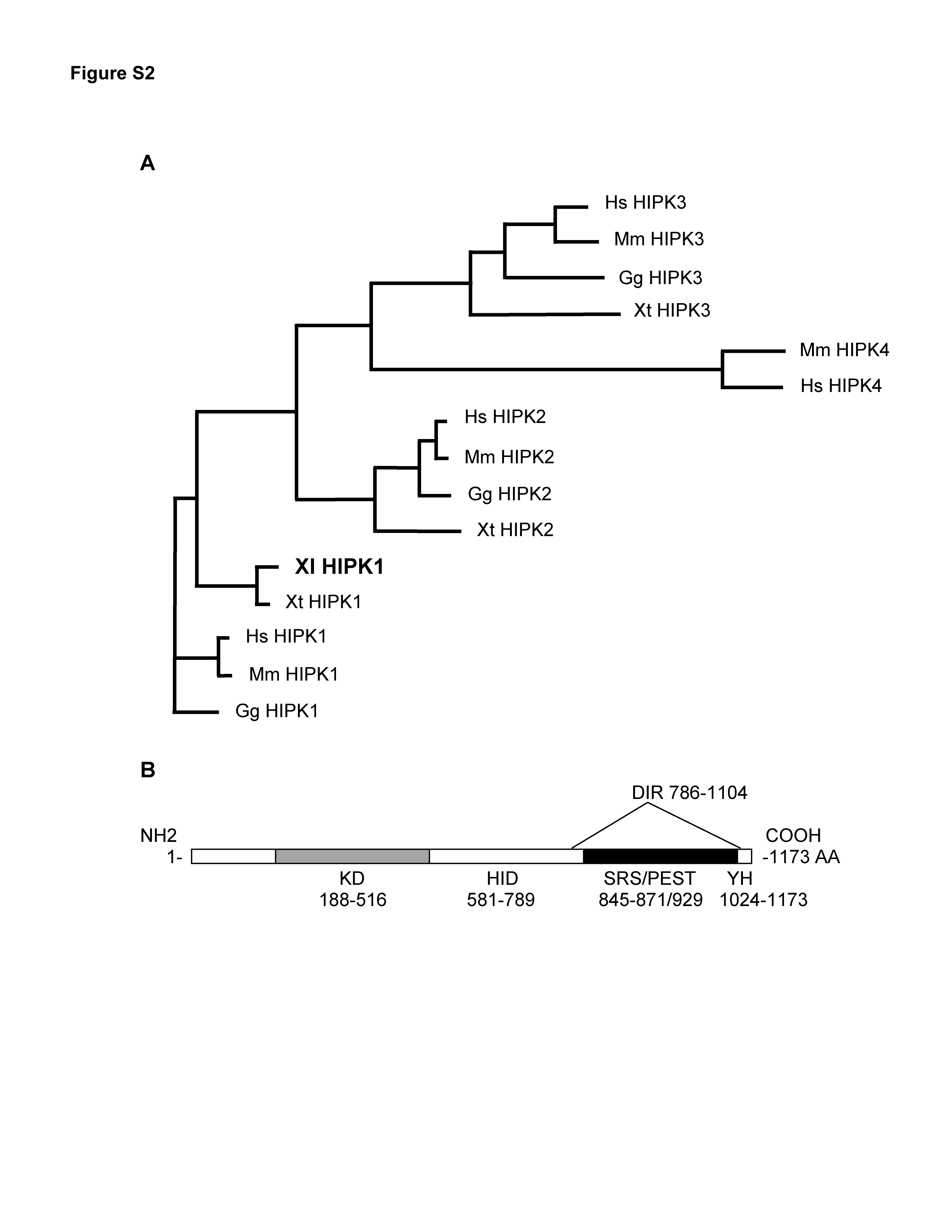

Supplement: Figure S2 — An X. laevis ortholog of Hipk1. (A) The X. laevis cDNA identified in this study is most similar to Hipk1 orthologs from other species, as opposed to other members of the Hipk gene family such as Hipk2, Hipk3, or Hipk4. (B) The primary sequence of X. laevis hipk1 is 1173 amino acids long and contains elements conserved in other family members including: a kinase domain (KD) with both Serine/Threonine and Tyrosine kinase consensus motifs, a homeodomain interaction domain (HID), a speckle retention sequence (SRS) and overlapping PEST domain, and a tyrosine-/histidine-rich (YH) domain. The extent of the clone from the yeast-2-hybrid screen is designated as the DIR (Dsh Interacting Region), corresponding to residues 786–1104. (0.66 MB TIF) [file pone.0004310.s002.tif]

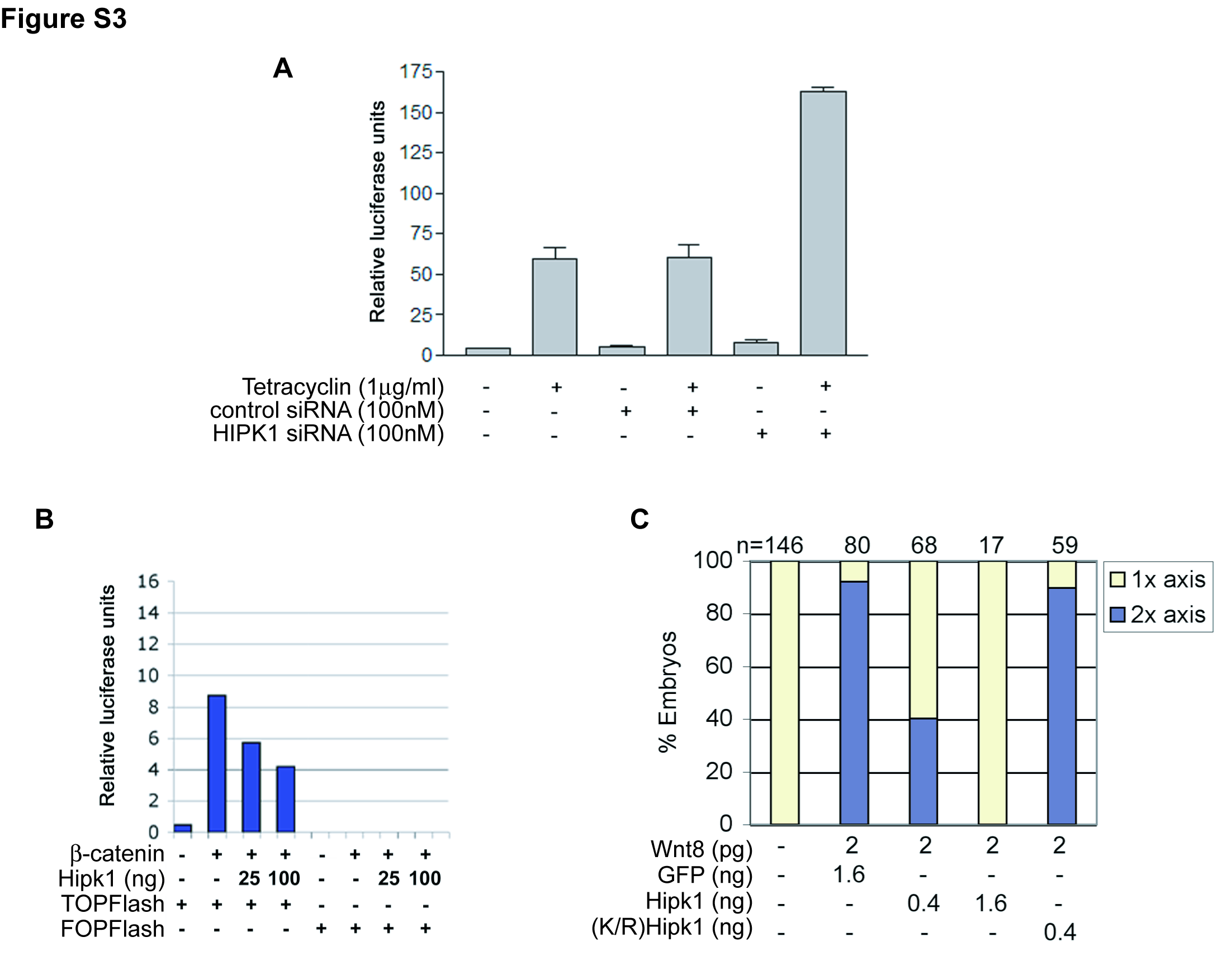

Supplement: Figure S3 — Knock-down of HIPK1 potentiates WNT1 activity in a tetracycline-inducible cell line, whereas over-expression of Hipk1 antagonizes Wnt/β-catenin signaling. (A) SiRNA directed against human HIPK1 potentiates the response of a β-catenin-responsive reporter to human Wnt1 expressed from a tetracycline-inducible transgene (See also Materials and Methods). (B) Transfection of HEK293 cells with plasmid encoding the X. laevis Hipk1 ortholog inhibits activation of a β-catenin-responsive reporter (TOPFlash) by β-catenin in a dose-responsive manner. (C) X. laevis axis duplication assay. Co-injection of Hipk1, but not (K/R)Hipk1 or GFP synthetic RNAs, interferes with duplication of the embryonic axis induced by Wnt8. (0.94 MB TIF) [file pone.0004310.s003.tif]

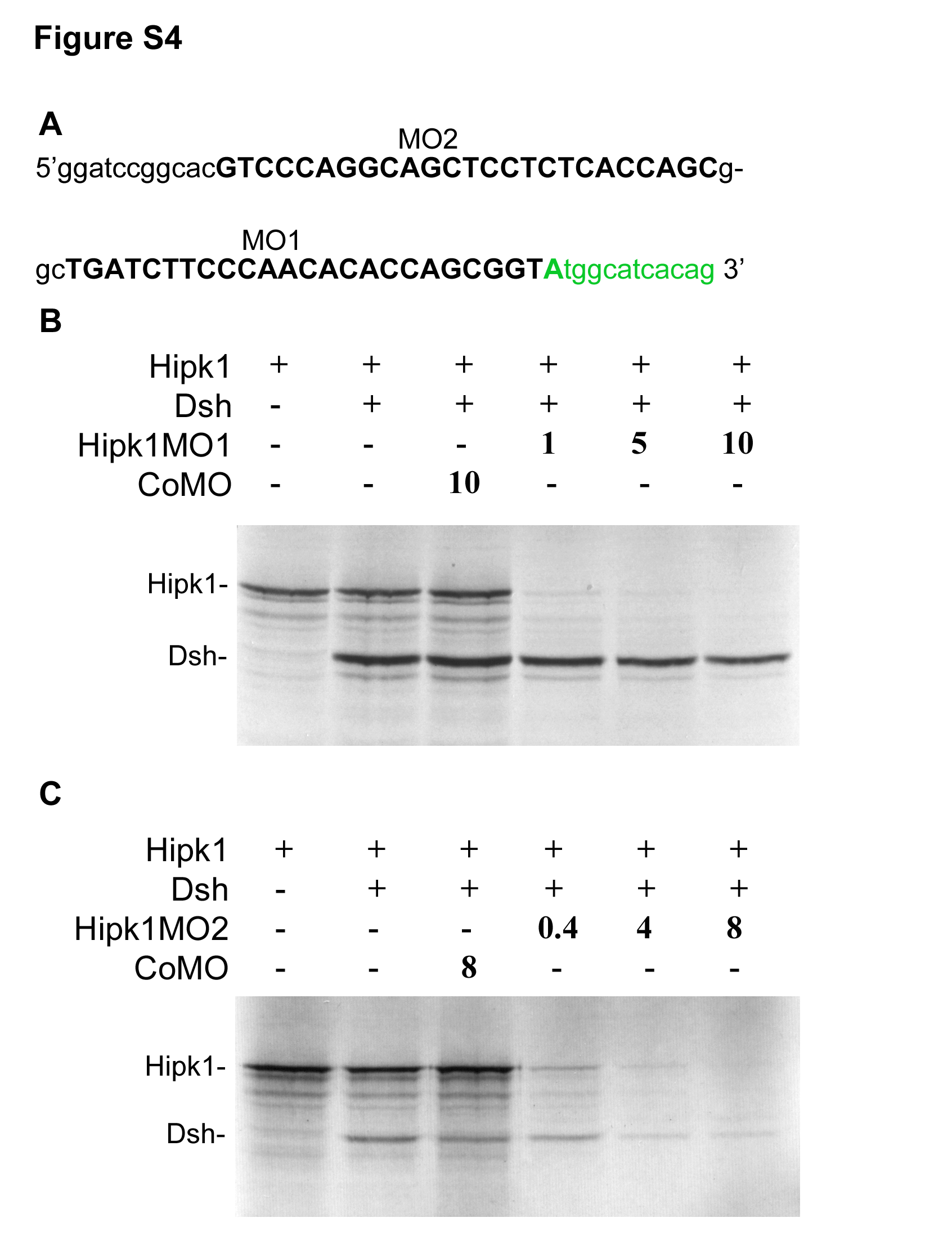

Supplement: Figure S4 — Hipk1 morpholinos specifically reduce translation of Hipk1. (A) Target sequences of Hipk1MO1 and Hipk1MO2 in the 5′UTR of X. laevis hipk1. (B, C) In vitro reticulocyte lysate transcription and translation reactions performed in the presence of 35S with plasmids encoding X. laevis Hipk1 and Dsh proteins; products separated by SDS-PAGE. Both Hipk1MO1 and Hipk1MO2 reduce Hipk1 protein in a dose-dependent manner. Based on its superior specificity as judged by less off-target effects on levels of the Dsh protein, Hipk1MO1 (B) was primarily used for Hipk1 loss-of-function experiments. Hipk1MO2 (C) was used at lower doses to corroborate findings obtained with Hipk1MO1. (1.21 MB TIF) [file pone.0004310.s004.tif]

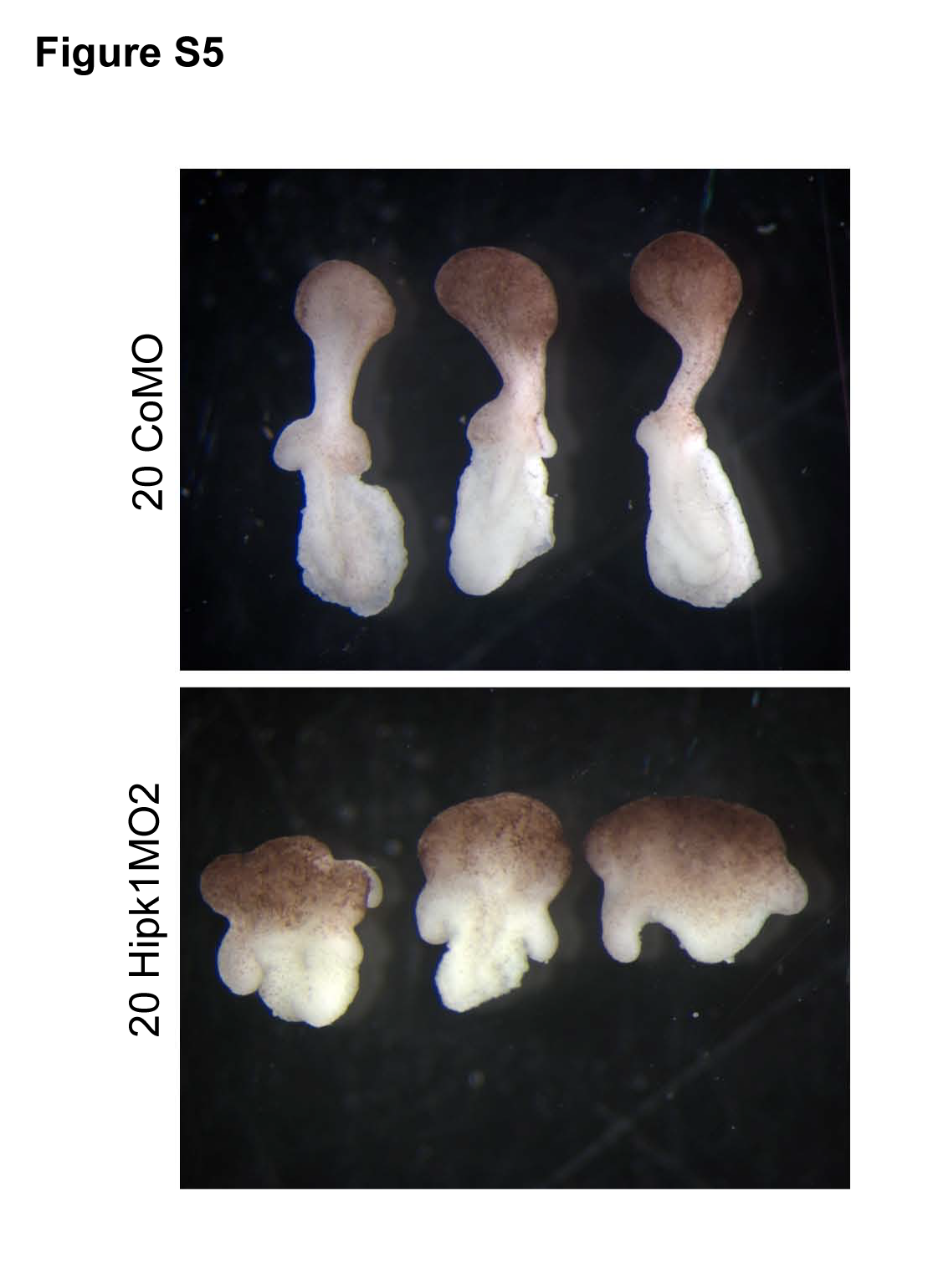

Supplement: Figure S5 — Hipk1MO2 inhibits Keller explant elongation. 20 ng per embryo of the control morpholino (CoMO) or Hipk1MO2 were injected into the DMZ. Keller explant sandwiches were prepared and cultured under glass until Stage 19 as described for Figure 6B. (5.13 MB TIF) [file pone.0004310.s005.tif]
