# Supplementary material for: Modulation of the β-Catenin Signaling Pathway by the Dishevelled-Associated Protein Hipk1
Source: PLoS One. 2009 Feb 2;4(2):e4310. doi: 10.1371/journal.pone.0004310 (PMC2629544; doi:10.1371/journal.pone.0004310)
Supplement: Table S1 — Supplemental Table 1 lists results for Hipk1 mRNA injections into the DMZ of X. laevis embryos. (0.02 MB PDF) [file pone.0004310.s006.pdf]

Supplemental Table 1. DMZ mRNA injection statistics

Condition	ng/embryo	% Full defect	% Partial defect	% Normal	Total #*
βgal mRNA	1.6	2	0	98	46
Hipk1 mRNA	0.4	4	8	88	48
Hipk1 mRNA	0.8	11	24	64	45
Hipk1 mRNA	1.6	57	23	20	44

*total from one representative experiment

Legend
ng/embryo = nanograms of reagent injected per embryo
% Full defect = percent of embryos with no neural fold fusion along anterior/posterior axis
% Partial defect= percent of embryos with partially fused neural folds along axis
% Normal = percent of embryos with normally fused neural folds along entire axis
Total # = number of embryos surviving past gastrulation
