# Supplementary material for: Modulation of the β-Catenin Signaling Pathway by the Dishevelled-Associated Protein Hipk1
Source: PLoS One. 2009 Feb 2;4(2):e4310. doi: 10.1371/journal.pone.0004310 (PMC2629544; doi:10.1371/journal.pone.0004310)
Supplement: Table S2 — Supplemental Table 2 lists results for Hipk1 morpholino injections into the DMZ of X. laevis embryos (0.02 MB PDF) [file pone.0004310.s007.pdf]

Supplemental Table 2. DMZ morpholino injection statistics

Condition	ng/embryo	% Full defect	% Partial defect	% Normal	Total #*
Uninjected	0	0	0	100	179
CoMO	80	3	2	95	457
Hipk1MO1	20	22	30	48	179
Hipk1MO1	40	70	19	11	253
Hipk1MO1	80	71	26	3	75
Uninjected	0	0	1	99	232
CoMO	32	7	7	86	136
Hipk1MO2	8	4	18	78	120
Hipk1MO2	16	39	23	38	121
Hipk1MO2	32	72	19	9	127

*combined total from 3 independent experiments

Legend
ng/embryo = nanograms of reagent injected per embryo
% Full defect = percent of embryos with no neural fold fusion along anterior/posterior axis
% Partial defect= percent of embryos with partially fused neural folds along axis
% Normal = percent of embryos with normally fused neural folds along entire axis
Total # = number of embryos surviving past gastrulation
